# Permanent Porosity in Hydroxamate Titanium–Organic Polyhedra

**DOI:** 10.1021/jacs.1c09278

**Published:** 2021-12-08

**Authors:** Belén Lerma-Berlanga, Javier Castells-Gil, Carolina R. Ganivet, Neyvis Almora-Barrios, Javier González-Platas, Oscar Fabelo, Natalia M. Padial, Carlos Martí-Gastaldo

**Affiliations:** † Functional Inorganic Materials Team, Instituto de Ciencia Molecular (ICMol), Universitat de València, 46980 Paterna, València, Spain; ‡ Departamento de Física, Instituto Universitario de Estudios Avanzados en Física Atómica, Molecular y Fotónica (IUDEA), MALTA Consolider Team, Universidad de La Laguna, Avda. Astrofísico Fco. Sánchez s/n, 38204 La Laguna, Tenerife, Spain; § Institut Laue Langevin, 71 avenue des Martyrs, CS 20156, 38042 Grenoble Cedex 9, France

## Abstract

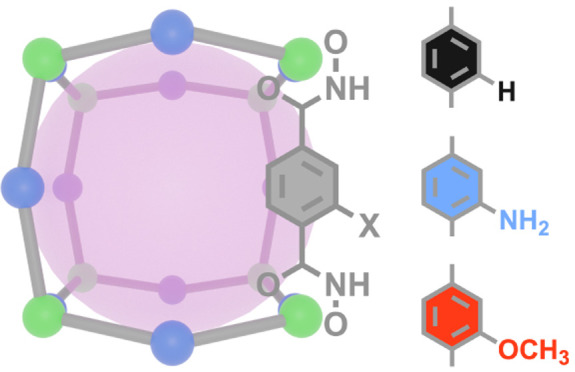

Following the synthesis of hydroxamate titanium–organic frameworks, we now extend these siderophore-type linkers to the assembly of the first titanium–organic polyhedra displaying permanent porosity. Mixed-linker versions of this molecular cage (cMUV-11) are also used to demonstrate the effect of pore chemistry in accessing high surface areas of near 1200 m^2^·g^–1^.

Metal–organic polyhedra (MOPs) are hybrid molecular complexes assembled by coordination linkages.^1^ These supramolecular cages feature intrinsic molecular porosity, which makes them attractive in host–guest recognition,^2,3^ separation,^4,5^ storage,^6^ and catalysis.^7^ Compared with extended porous solids such as metal–organic frameworks (MOFs), covalent organic frameworks (COFs), and zeolites, MOPs are relatively unexplored and represent a rapidly growing class of porous molecular solids.^8^ Their molecular nature can ease their processability for integration into membranes^9^ or synthetic channels.^10^ However, permanent porosity in MOPs is still less common because of the difficulties in designing robust cages that display porosity after guest removal. Even though the assembly of cages with prefabricated porosity can be controlled with the symmetry and directionality of metal–organic nodes,^11^ the accessible porosity also depends on their packing in the solid state, which is controlled by intermolecular interactions that are much weaker than the directional bonding in extended networks.

This is evidenced by the small number of surface areas measured for metal–organic cages.^8^ Among the 120 Brunauer–Emmett–Teller (BET) surface areas reported since 2005,^12^ only 10 reach the 1000 m^2^·g^–1^ limit up to the maximum value of 1320 m^2^·g^–1^ reported in 2019.^13^ Analysis of these examples reveals the predominance of cuboctahedral (*cuo*) and octahedral (*oct*) cages assembled from bent dicarboxylic acid ligands and bimetallic paddlewheel units. Like the case of extended reticular solids, further advancement of the field will be fueled by expanding the toolbox of organic linkers, metals, and node geometries for the assembly of robust porous polyhedra. Close to 90% of the MOPs reported are based on clusters with high nuclearity reminiscent of the secondary building units in MOFs. Even though the design of mononuclear cages is common in supramolecular chemistry, they hardly display permanent porosity. This problem is associated with the use of soft N-donor linkers that render weak metal–linker joints more prone to collapse. The use of higher-p*K*_a_ imidazolates as connectors has proven to be successful in directing the assembly of porous cages with In^3+^ (MOC-2)^14^ and Pd^2+^ (MOP-100).^15^ We argued that this same concept could be extended to polycarboxylate linkers by replacing carboxylic groups with hydroxamic groups. This siderophore-type chelating agent combines coordination modes similar to those for carboxylate with stronger bonds with some transition metal ions. We recently reported the formation of a porous titanium–organic framework by the use of benzene-1,4-dihydroxamic acid (*p*-H_4_bdha).^16^ MUV-11 displayed excellent chemical stability as result of the formation of octahedral Ti(IV) mononuclear chelates. This same chelate was also used by Tezcan to design a 3D framework built from exceptionally stable [Fe_4_(*m*-H_2_bdha)_6_] nodes,^17^ reminiscent of the hydroxamate supramolecular cages first reported by Raymond with Fe(III).^18^ Also, the use of highly charged metals such as Zr or Ti(IV) is accepted as an ideal choice for the assembly of chemically stable cages from robust linkages.^19^

We investigate the use of hydroxamate in the assembly of titanium–organic cages. cMUV-11 (cMUV = cage-type Material of Universidad de València) is the first example of a titanium MOP displaying permanent porosity. cMUV-11 was synthesized as dark-red octahedral crystals with sizes near 100 μm by the reaction of titanium(IV) isopropoxide and *p*-H_4_bdha in *N*,*N*-dimethylformamide (DMF) with benzoic acid as a modulator (Figure 1a). Compared with the synthesis of MUV-11,^16^ the use of shorter reaction times, milder temperatures, and benzoic acid as a modulator are important to avoid the formation of the extended framework. Single-crystal X-ray diffraction analysis revealed that cMUV-11 crystallizes in the tetragonal space group *I*4/*m* (*a* = 24.06 Å, *c* = 24.70 Å). The structure is based on discrete neutral cubes with formula [Ti_8_(*p*-H_2_bdha)_8_(*p*-bdha)_4_] and Ti···Ti diagonals of 18.896 Å (Figure 1b). Like MUV-11, one-third of the hydroxamic −NH groups in the linkers are deprotonated for the assembly of a neutral cage. Eight single-node titanium connection points with links at an angle of near 105° (η) sit in the vertices and are linked by 12 *p*-bdha linkers located in the edges and bent with an angle of θ = 157°. This conforms to a distorted cube (*cub*) that deviates from the ideal 90° dihedral angles of the regular hexahedron. This is imposed by the five-membered chelate ring formed by the hydroxamate group that distorts the internal angles of the TiL_3_ octahedra. This configuration seems to be key for the assembly of the structure, as it reinforces the rigidity of the nodes and locks the hydroxamate groups in place to enable intermolecular hydrogen bonds. The two vertices of the cube edges interact with eight neighboring cages by following a vertex-to-vertex pattern. All of the N–H···O hydrogen bonds (*d*_N–O_ = 2.624 Å) are symmetry-equivalent and involve complementary hydroxamic groups from adjacent edges that act as donors and acceptors (Figure 1c). As result, the cages pack in a 3D open framework with a *pcb* topology (Figure 1d) and microporous cavities intrinsic to the *cub* cages. The analysis of the crystallographic file reveals pore windows of 0.7 nm and internal cavities of 1.4 nm, giving a solvent-accessible volume of near 70%.

**Figure 1:**
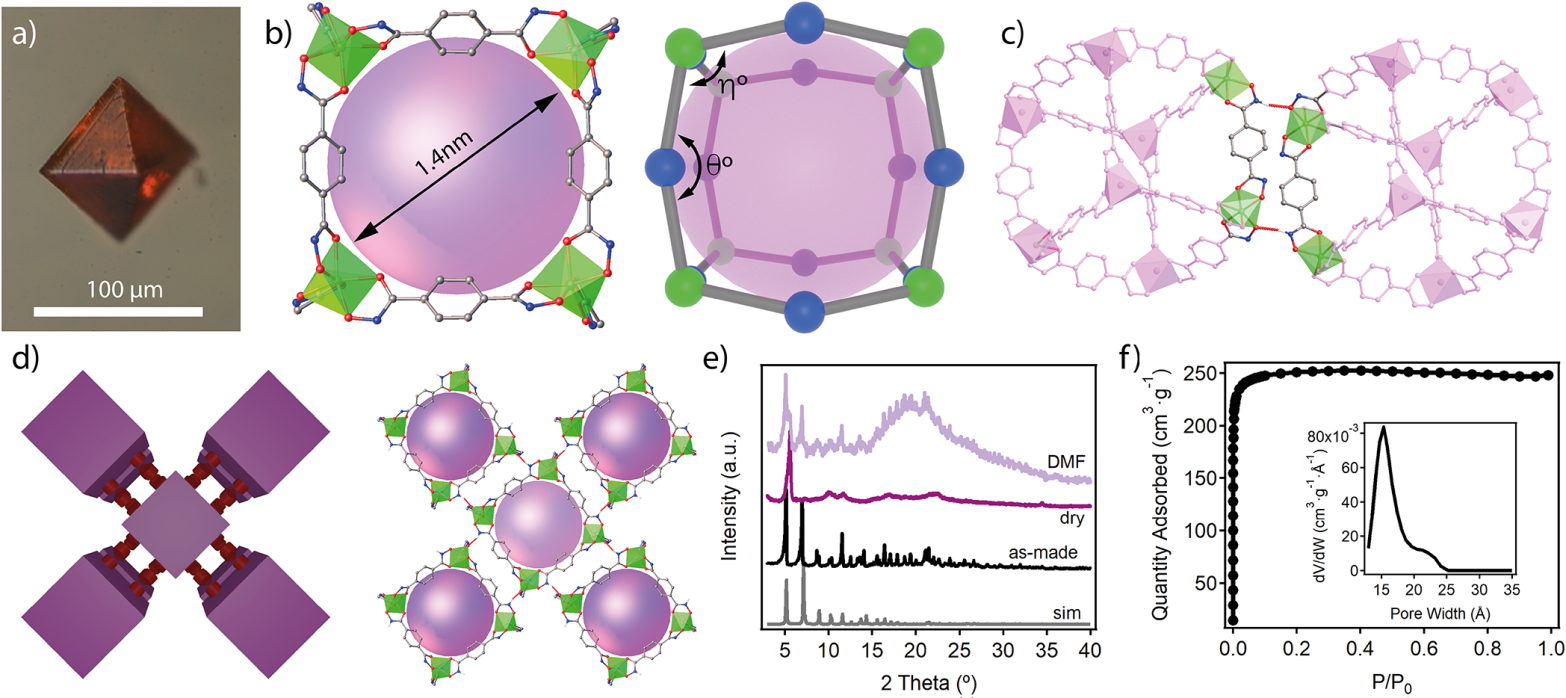
(a) Crystal of cMUV-11. (b) Structure and microporous cavity of the titanium cages featuring a slightly distorted cube geometry and internal cavities of 1.4 nm. (c) Hydrogen-bonding interactions between hydroxamate groups from adjacent cages. (d) Vertex-to-vertex packing of the cages into a 3D open framework. (e) Structural response to evacuation and exposure to DMF. (f) N_2_ isotherm and PSD at 77 K after acetone exchange.

As summarized in Supporting Information (SI) section S4, scanning electron microscopy (SEM) and LeBail refinement of the powder X-ray diffraction (PXRD) data for bulk samples were used to confirm the phase purity. Thermogravimetric analysis (TGA) in air showed well-defined decomposition steps at 200 and 430 °C that correspond to the early oxidation of the linker followed by cage decomposition. This thermal stability is identical to that of MUV-11^16^ and comparable to those of other Ti^4+^ frameworks,^20,21^ suggesting that the thermal stability is not affected by the reduction in dimensionality. We tested the chemical stability of the cage by soaking freshly made crystals in water for 24 h followed by Inductively Coupled Plasma-Mass Spectrometry (ICP-MS) analysis of the supernatant. Metal leaching was almost negligible (4 mg·L^–1^), confirming the ability of hydroxamate chelates to provide excellent resistance toward hydrolysis. Our preliminary tests suggest that cMUV-11 displays limited solubility in conventional solvents. We next examined the structural stability of cMUV-11 by evacuating the crystals in vacuum at room temperature. As shown in Figure 1e, this treatment induced a drastic broadening of the diffraction lines indicative of partial collapse of the structure. Still, we were able to identify (110), (220), and (310) diffraction lines from the original structure. The original diffraction pattern was recovered by immersion of the crystal in DMF, confirming the flexibility of the hydrogen bonds that control the cage packing and anticipating the importance of finding an adequate activation protocol to prevent structural collapse. We opted for solvent exchange with acetone followed by evacuation at 10^–3^ mbar at 40 °C for 16 h. The crystals displayed a reversible type-I isotherm characteristic of a microporous material, with no signature of hysteretic behavior and a BET surface area of 1020 m^2^·g^–1^ (Figure 1f). This value is not far from the highest porosity reported for a MOP. All examples displaying porosities above 1000 m^2^·g^–1^ to date are based on paddlewheel or multinuclear clusters, which are more likely to yield robust cages. In our case, the incorporation of rigid hydroxamate chelates seems to be crucial in controlling the response of cMUV-11 to evacuation and enable permanent porosity in a coordination cage based on mononuclear nodes. The experimental pore size distribution (PSD) calculated using nonlinear Density Functional Theory (DFT) methods shows a narrow peak centered at 1.5 nm, in good agreement with the dimensions of the microporous cavity estimated from the crystallographic analysis. There is also a broader peak at 2.2 nm that accounts for 10% of the porosity. This extrinsic mesoporosity cannot be directly correlated with the structure of cMUV-11 and might be indicative of changes in the cage packing upon evacuation. To better understand this behavior, we explored the use of other volatile solvents such as ether and hexane by following the same evacuation protocol. We observed a reduction in the surface area in both cases, down to a minimum of 750 m^2^·g^–1^ for hexane. This change is concomitant with a decrease in the contribution of the intrinsic micropores to the global porosity at the expense of an increase in the extrinsic mesoporosity.

Controlling the cage structure and connectivity for more stable crystalline arrangements is certainly essential to optimize the porosity.^22^ We hypothesized that this might be investigated in our case by using *p*-bdha linkers functionalized with complementary hydrogen-bond donor (D) and acceptor (A) groups that might modify the connectivity pattern fixed by the hydroxamate groups in cMUV-11. We synthesized the mixed-linker cages cMUV-11-NH_2_ and -OCH_3_ by following the same protocol used for the original cage but using binary combinations of *p*-bdha with 2-aminobenzene-1,4-dihydroxamic acid (*p*-H_4_bdha-NH_2_) or 2-methoxybenzene-1,4-dihydroxamic acid (*p*-H_4_bdha-OCH_3_) at variable molar ratios ranging from 10 to 100% (Figure 2a). We used a robotic platform for the automated dosing of solutions to ensure maximum reproducibility. Multivariate cages were isolated as crystals with size, color, and morphology similar to those of the pristine material (Figure 2b,c). The experimental ratio of the linkers in cMUV-11-X% was analyzed with ^1^H NMR (Nuclear Magnetic Resonance) spectroscopy after digestion of the crystals in acid. Figure 2d shows the rates of incorporation of *p*-bdha-X into the crystals as functions of the percentage of linkers in solution. *p*-bdha-OCH_3_ follows a linear regime up to a maximum of near 70% from which the MOP cannot be formed. In turn, *p*-bdha-NH_2_ follows a sigmoidal trend with negligible incorporation at linker percentages below 30% followed by progressive incorporation at higher ratios up to 90%. This suggests a distinct effect of the linker in controlling the assembly of [Ti_8_(*p*-bdha)_12–*y*_(*p*-bdha-X)_*y*_] (X = NH_2_, OCH_3_) cages, possibly due to their different abilities to behave as hydrogen-bond donors or acceptors. The impact of −OCH_3_ (A) groups is only detrimental at higher concentrations. In turn, the presence of −NH_2_ (D/A) requires higher concentrations for the cage assembly. This effect is even more drastic when *p*-H_4_bdha is combined with 2-hydroxybenzene-1,4-dihydroxamic acid (*p*-H_4_bdha-OH), for which we do not observe the formation of any solid regardless of the relative ratio used. Our results highlight the effect of linker functionalization in controlling the formation and composition of multivariate cMUV-11-X cages. Also, single-component cages cannot be prepared from the functionalized linkers alone, suggesting that the functionalized linkers might destabilize cage assembly compared to *p*-bdha (SI section S2).

**Figure 2:**
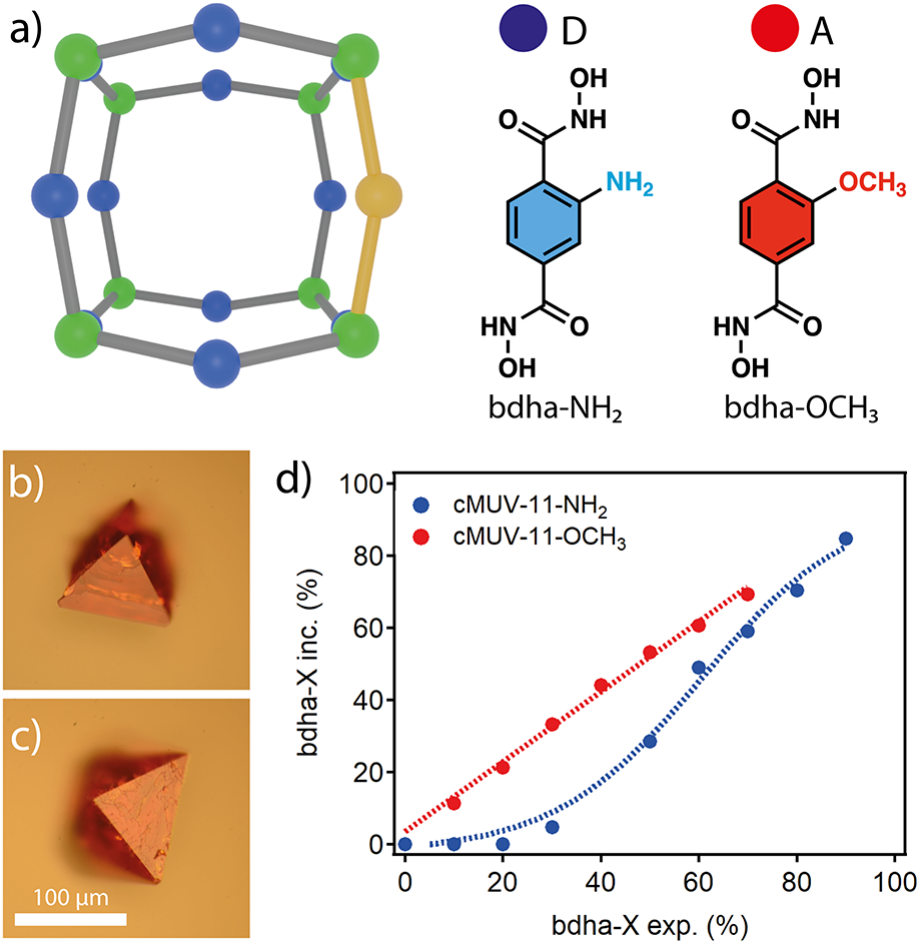
(a) Mixed-linker cages from combination of *p*-bdha with *p*-bdha-NH_2_ or -OCH_3_. (b, c) Crystals of (b) cMUV-11-NH_2_-50% and (c) -OCH_3_-50%. (d) Rate of incorporation of *p*-bdha-X linkers into the crystals as a function of their concentration in solution.

The phase purity and homogeneity of the samples were evaluated by PXRD and SEM, respectively (SI section S4), confirming the formation of mixed-linker phases isostructural to cMUV-11 in all cases. Single crystals of cMUV-11-NH_2_-50% and -OCH_3_-50% were measured at low temperature (100 K) to elucidated the effect of linker functionalization on the cage packing (Figure 3a,b). Both structures show local crystallographic disorder affecting the ortho and meta positions of the aromatic ring as result of the combination of −H and −NH_2_/–OCH_3_ groups, consistent with the ratios calculated by ^1^H NMR analysis. The incorporation of these groups prompts the formation of additional intracage N–H···N (−NH_2_) or N–H···O (−OCH_3_) interactions but does not change the network of intercage hydrogen bonds that control the cage packing and are slightly shortened by 0.01 and 0.03 Å, respectively. Our DFT calculations show that compared to cMUV-11, the effect of both substituents on the charge density around the O and N atoms of the hydroxamate group is negligible (Figure 3c). This is translated into minimum changes in the Ti–O bond distances and corresponding strengths of the linkages for similar thermal stabilities according to TGA. The ICP analysis also confirmed minimum metal leaching after 24 h. We argued that the main differences between the pristine and functionalized cages would be dominated by the changes in pore polarity. We analyzed the responses to solvent evacuation of cMUV-11-NH_2_-50% and -OCH_3_-50% after solvent exchange with acetone and hexane following the same protocol as used with cMUV-11. The PXRD patterns of the solids after evacuation show clear differences in their structural response. The introduction of polar -NH_2_ groups results in more drastic structural collapse after solvent removal, which becomes even more acute for a polar solvent such as acetone (Figure 3d). In turn, nonpolar methoxy groups seem better fitted to minimize solvent interactions and avoid disruption of the hydrogen-bonding network responsible for long-range packing. As result, the MOP retains better crystallinity regardless of the polarity of the solvent used. The effect of *p*-bdha-X is also translated into the accessible porosity of the multivariate cages calculated from N_2_ isotherms (Figure 3e). For the cMUV-11-NH_2_-X% series, only the samples exchanged with hexane and containing low concentrations of amine groups (≤20%) display permanent porosity with a maximum BET value of 620 m^2^·g^–1^ (Figure 3f). This value is below those displayed by cMUV-11 tested under the same conditions and confirms the detrimental effect of these groups in rigidifying the cage assembly toward solvent evacuation. In turn, −OCH_3_ functionalization below 50% yields surface areas of nearly 1200 or 1100 m^2^·g^–1^ after treatment with acetone or hexane, which are higher than those of cMUV-11, suggesting the effect of intercage interactions in modulating the permanent porosity in this family of MOPs. This is consistent with the irreversible collapse of the -H and -NH_2_ cages in water that can be reverted back only in the case of cMUV-11-OCH_3_.

**Figure 3:**
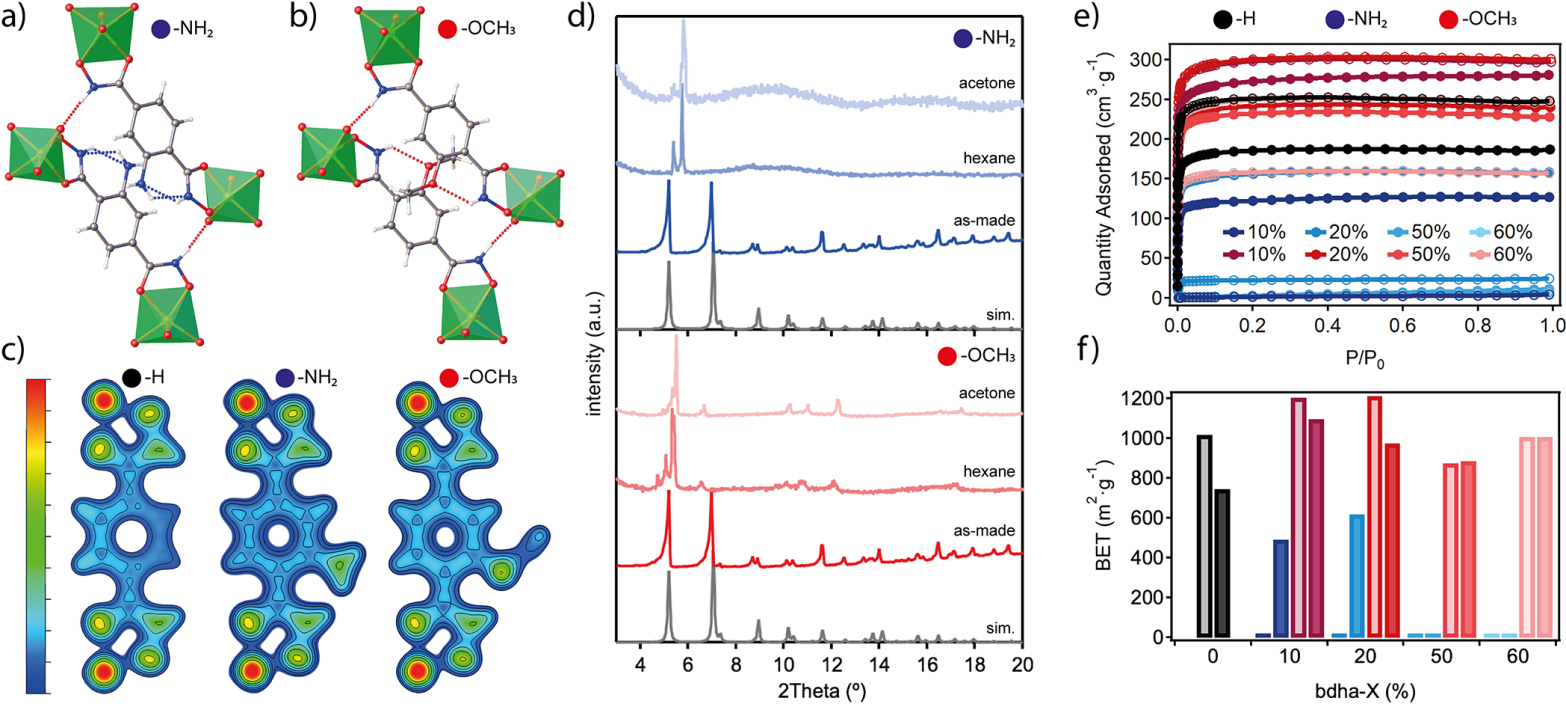
(a, b) Hydrogen-bonding interactions in (a) cMUV-11-NH_2_-50% and (b) -OCH_3_-50% crystals. (c) DFT calculations showing the charge density changes in the linker for the different substituents. (d) Changes in the structural response of both MOPs to solvent evacuation. (e) N_2_ isotherms at 77 K of cMUV-11-X% (X = 10, 20, 50, 60) cages exchanged with acetone (open symbols) or hexane (solid symbols) and (f) corresponding BET surface areas compared with MUV-11 in black.

cMUV-11 is the first example of a permanently porous titanium–organic cage. This molecular solid is packed from vertex-to-vertex hydrogen-bonded microporous hydroxamate mononuclear cubes that are compatible with linker functionalization. This synthetic versatility was used to produce mixed-linker cages with tailorable pore chemistry and varying sensitivity to solvent evacuation for permanent porosities above 1000 m^2^·g^–1^. Compared with extended titanium MOFs, which are simultaneously treated as semiconductor (TiO_2_) or molecular catalysts,^23^ we are confident that these titanium MOPs might be an ideal platform to engineer photocatalytic performance in porous solids by using only molecular concepts exclusive of homogeneous catalysis.

## Supporting Information Available

Synthetic and experimental details, physical characterization, and supporting tables and figures (PDF)

## Accession Codes

CCDC 2106822–2106824 contain the supplementary crystallographic data for this paper. These data can be obtained free of charge via www.ccdc.cam.ac.uk/data_request/cif, or by emailing data_request@ccdc.cam.ac.uk, or by contacting The Cambridge Crystallographic Data Centre, 12 Union Road, Cambridge CB2 1EZ, U.K.; fax: +44 1223 336033.
