# Supplementary material for: Permanent Porosity in Hydroxamate Titanium–Organic Polyhedra
Source: J Am Chem Soc. 2021 Dec 8;143(50):21195–9. doi: 10.1021/jacs.1c09278 (PMC9157491; doi:10.1021/jacs.1c09278)
Supplement: Supplementary file 1 — ja1c09278_si_001.pdf [file ja1c09278_si_001.pdf]

Supplementary Information

Permanent porosity in hydroxamate titanium-organic polyhedra

Belén Lerma-Berlanga,† Javier Castells-Gil,† Carolina R. Ganivet,† Neyvis Almora-Barrios,† Javier González-Platas,§ Oscar Fabelo,⊥ Natalia M. Padial† and Carlos Martí-Gastaldo†*

† Functional Inorganic Materials Team, Instituto de Ciencia Molecular (ICMol), Universitat de València, Paterna 46980, València, Spain.

§ Departamento de Física. Instituto Universitario de Estudios Avanzados en Física Atómica, Molecular y Fotónica (IUDEA). MALTA Consolider Team. Universidad de La Laguna, Avda. Astrofísico Fco. Sánchez s/n, La Laguna, Tenerife, E-38204, Spain.

⊥ Institut Laue Langevin, 71 avenue des Martyrs, CS 20156, Grenoble, Cedex 9 38042, France.

Table of contents

Supplementary Information 1

S1. General considerations: starting materials and characterization. 3

Materials and reagents 3

Physical and chemical characterization 3

S2. Synthesis of cMUV-11 4

S2.1. Synthesis of organic ligands 4

Preparation of 1,4-benzo-dihydroxamic acid (*p*-H_4bdha) 4

Preparation of 1,4-benzo-2-amine-dihydroxamic acid (*p*-H_4bdha-NH_2) 4

Preparation of 1,4-benzo-2-methoxy-dihydroxamic acid (*p*-H_4bdha-OCH_3) 6

Preparation of 1,4-benzo-2-hydroxy-dihydroxamic acid (*p*-H_4bdha-OH) 9

S2.2. Synthesis of cMUV-11 11

S2.3. Synthesis of cMUV-11-NH_2 & cMUV-11-OCH_3 11

S3. cMUV-11 structures. Single-Crystal X-ray Diffraction Analyses 16

S4. Chemical characterization 20

S4.1. cMUV-11 20

Scanning Electron Microscopy (SEM) 20

Thermogravimetric Analysis (TGA) 20

Powder X-Ray Diffraction (PXRD) 21

S4.2. Solubility test of cMUV-11 22

S4.3. cMUV-11-NH_2 & cMUV-11-OCH_3 25

Scanning Electron Microscopy (SEM) 25

Thermogravimetric Analysis (TGA) 26

Elemental analysis (EA) 26

Powder X-Ray Diffraction (PXRD) 27

S5. Activation of cMUV-11 cages and N_2 adsorption 28

N_2 isotherms 28

S6. Chemical and Structural Stability 32

ICP-MS measurements 32

Powder X-Ray Diffraction (PXRD) after water incubation 33

CO_2 adsorption after water incubation 34

Scanning Electron Microscopy (SEM) after water incubation 35

S7. Computational calculations 36

References 37

S1. General considerations: starting materials and characterization.

Materials and reagents

Dimethyl 2-aminoterephthalate, dimethyl terephthalate, hydroxylamine hydrochloride ($NH_2OH{\cdot}HCl$, 99 %), sodium hydroxide (NaOH ≥97.0%, pellets), iodomethane (CH_3I, ≥99.0% (GC)) potassium carbonate (≥99.0%) and anhydrous *N,N*-Dimethylformamide (DMF, 99.8 %) were purchased from Sigma-Aldrich. 2-hydroxy-terephthalic acid dimethyl ester was purchased from Fluorochem. Methanol, acetone, hexane, and ether (≥ 99.9%) were purchased from Scharlab. Ultrapure water from Milli-Q equipment was used when required. All reagents and solvents were used without any previous purification unless specified.

Physical and chemical characterization

- Elemental analysis (EA): Carbon, nitrogen and hydrogen contents were determined by microanalytical procedures using a LECO CHNS.

- Thermogravimetric analysis (TGA) were carried out with a Mettler Toledo TGA/SDTA 851 apparatus between 25 and 600 °C under ambient conditions (10 $°C{\cdot}min^{-1}$ scan rate and an air flow of 30 $mL{\cdot}min^{-1}$).

- Nuclear magnetic resonance (NMR) spectra were recorded on Bruker DRX-500 spectrometer and were calibrated to the residual solvent peak (DMSO-d_6 at 2.50 ppm ^{1}H-NMR). The following abbreviations were used to explain multiplicities: s = singlet, d = doublet, t = triplet, q = quartet, m = multiplet, br = broad.

- Powder X-Ray Diffraction (PXRD) patterns were collected in a PANalytical X'Pert PRO diffractometer using copper radiation (Cu Kα = 1.5418 Å) with an X'Celerator detector, operating at 40 mA and 45 kV. Profiles were collected in the 2° < 2θ < 40° range with a step size of 0.017°.

- Scanning Electron Microscopy (SEM): particle morphologies and dimensions were studied with a Hitachi S4800 scanning electron microscope at an accelerating voltage of 20 kV, over metalized samples with a mixture of gold and palladium for 90 seconds.

- Inductively coupled plasma mass spectrometry (ICP-MS): the measurements were carried out with an Agilent 7900 apparatus.

- Gas adsorption measurements were recorded on a Micromeritics 3Flex apparatus at relative pressures up to 1 atm. The samples were exchanged with low boiling point solvent and degassed overnight at 40 °C and 10^{-6} Torr prior to analysis. Surface area, pore size and volume values were calculated from N_2 adsorption-desorption isotherms (77 K) Specific surface area was calculated by multi-point Brunauer-Emmett-Teller (BET) method. Total pore volume was taken at P/P_0=0.96. Pore size distribution was analysed by using the solid density functional theory (NLDFT) for the adsorption branch by assuming a cylindrical pore model.

S2. Synthesis of cMUV-11

S2.1. Synthesis of organic ligands

Preparation of 1,4-benzo-dihydroxamic acid (*p*-H_4bdha)

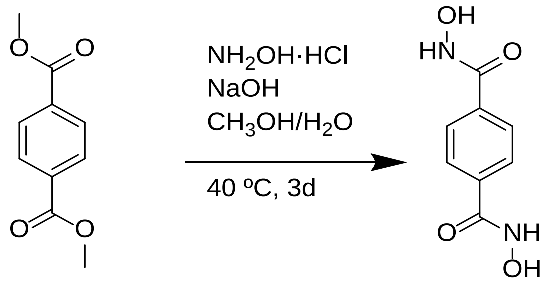

Scheme S1. Synthesis of *p*-H_4bdha ligand.

The synthesis of *p*-H_4bdha was carried out according to the procedure described by Marmion[1] with minor modifications reported by our group.[2]

Preparation of 1,4-benzo-2-amine-dihydroxamic acid (*p*-H_4bdha-NH_2)

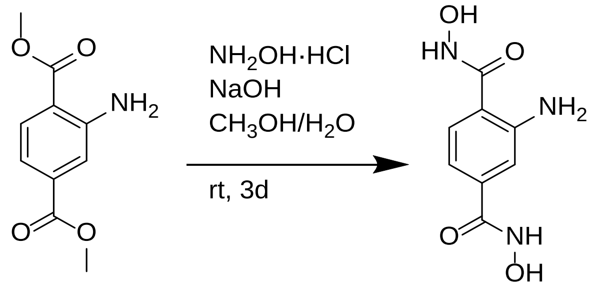

Scheme S2. Synthesis of *p*-H_4bdha-NH_2 ligand.

The synthesis of *p*-H_4bdha-NH_2 was carried out following the procedure for *p*-H_4bdha with slight modifications. Hydroxylamine hydrochloride (1.80 g, 25.9 mmol) was mixed with sodium hydroxide (2.06 g, 51.5 mmol) in deionized water (13 mL). The solution was then added to a suspension of dimethyl 2-aminoterephthalate (1.79 g, 8.6 mmol) in methanol (15 mL). The resulting mixture was stirred for 72 hours at room temperature and subsequently acidified to pH 6 with a solution of 30 % acetic acid. The yellow solid was filtered and washed with deionized H_2O. The desired solid *p*-H_4bdha-NH_2 was dried under reduced pressure overnight (89 % yield). ^{1}H-NMR (300 MHz, DMSO-d_6) δ: 7.34 (d, J = 8.1 Hz, 1H), 7.10 (d, J = 1.5 Hz, 1H), 6.79 (dd, J = 8.1, 1.6 Hz, 1H). ^{13}C-NMR (75 MHz, DMSO-d_6) δ: 166.0 (C), 149.0 (C), 135.7 (C), 127.6 (CH), 115.0 (CH), 112.6 (CH).

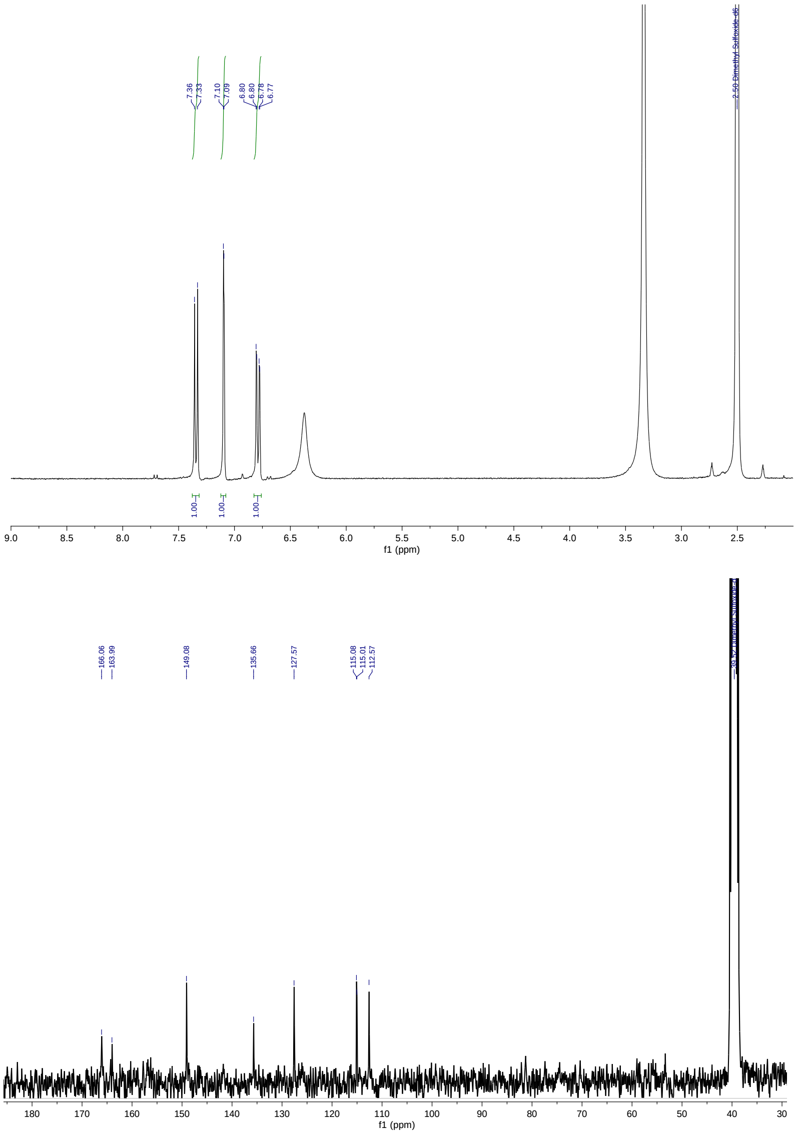

Figure S1. ^{1}H-NMR spectrum of compound *p*-H_4bdha-NH_2 in DMSO-d_6 (top) and ^{13}C-NMR spectrum of compound *p*-H_4bdha-NH_2 in DMSO-d_6 (bottom).

Preparation of 1,4-benzo-2-methoxy-dihydroxamic acid (*p*-H_4bdha-OCH_3)

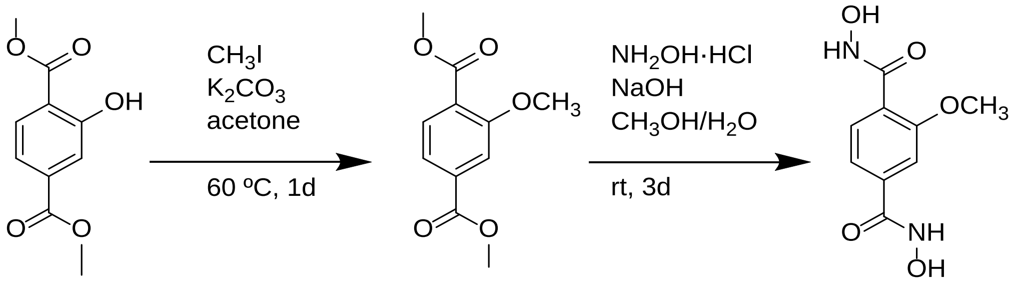

Scheme S3. Synthesis of *p*-H_4bdha-OCH_3 ligand.

The first step was the hydroxy group methylation, that was carried out according to the method reported by Min Kim and co-workers.[3] 2-Hydroxy-terephthalic acid dimethyl ester (1.65 g, 7.8 mmol), potassium carbonate (11.04 g, 78.4 mmol) and iodomethane (2.44 mL, 39.2 mmol) were charged in 30 mL of acetone. The mixture was stirred and heated at 60 °C for 24 h. After this time, the organic layer was extracted with dichloromethane and H_2O. The organic layer was dried over $MgSO_4$ and evaporated to obtain the pure product as a white solid (bdMe_2-OCH_3). ^{1}H-NMR (300 MHz, DMSO-*d6*) δ: 7.74 (d, J = 8.3 Hz, 1H), 7.61 - 7.57 (m, 2H), 3.89 (s, 6H), 3.81 (s, 3H). ^{13}C-NMR (75 MHz, DMSO-*d6*) δ: 165.7 (C), 165.5 (C), 157.7 (C), 133.7 (C), 130.7 (CH), 124.5 (C), 120.8 (CH), 112.5 (CH), 56.0 (CH_3), 52.5 (CH_3), 52.2 (CH_3).

Then, a solution of hydroxylamine hydrochloride (1.62 g, 23.3 mmol) and sodium hydroxide (1.86 g, 46.5 mmol) in water (19 mL) was added to a suspension of dimethyl 2-methoxyterephthalate (bdMe_2-OCH_3) (1.73 g, 8.6 mmol) in methanol (19 mL). The resulting mixture was stirred for 72 hours at room temperature and subsequently acidified to pH 6 with a solution of 30 % acetic acid. The white solid was filtered and washed with deionized H_2O. The desired solid *p*-H_4bdha-OCH_3 was dried under reduced pressure overnight (90 % yield). ^{1}H-NMR (300 MHz, DMSO-*d6*) δ: 11.32 (brs), 10.72 (brs), 9.15 (brs), 7.57 (d, J = 7.8 Hz, 1H), 7.44 – 7.35 (m, 2H), 3.87 (s, 3H). ^{13}C-NMR (75 MHz, DMSO-*d6*) δ: 162.5 (C), 156.4 (C), 135.7 (C), 129.7 (CH), 125.1 (C), 118.8 (CH), 110.1 (CH), 55.8 (CH_3).

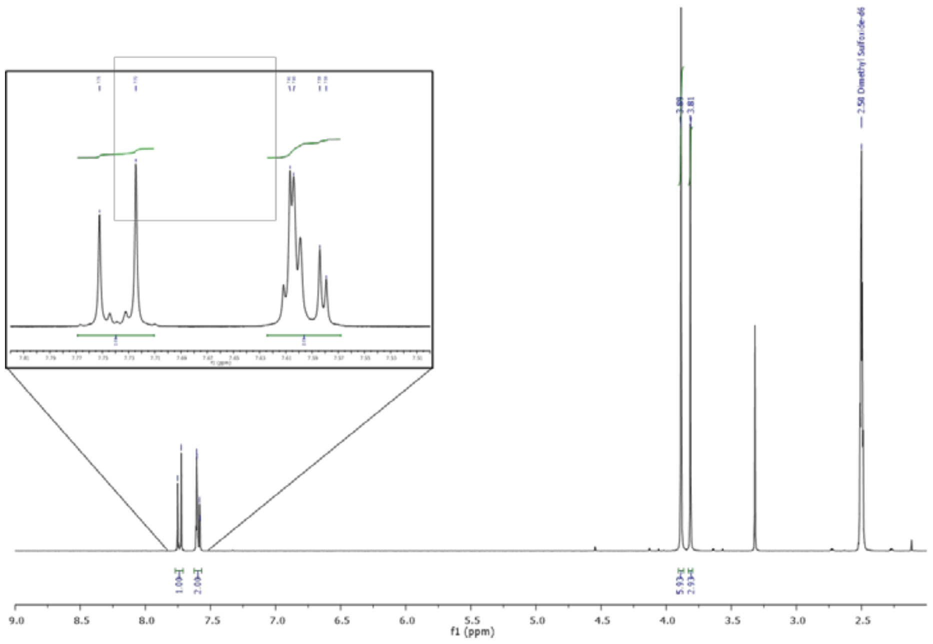

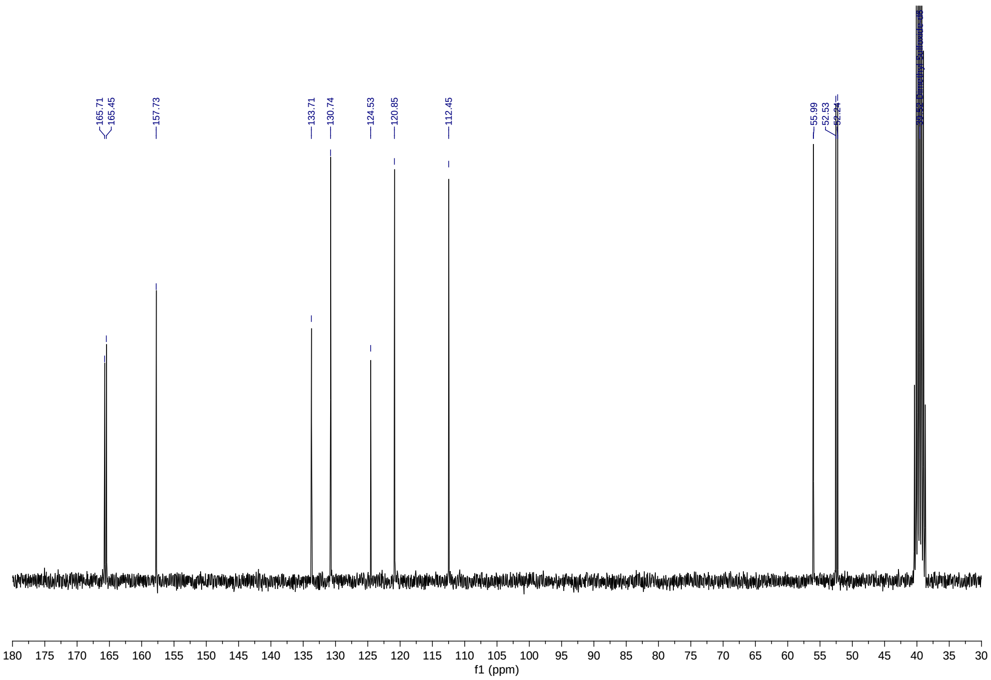

Figure S2. ^{1}H-NMR spectrum of compound bdMe$_2$-OCH$_3$ in DMSO-d_6 (top) and ^{13}C-NMR spectrum of compound bdMe$_2$-OCH$_3$ in DMSO-d$_6$ (bottom).

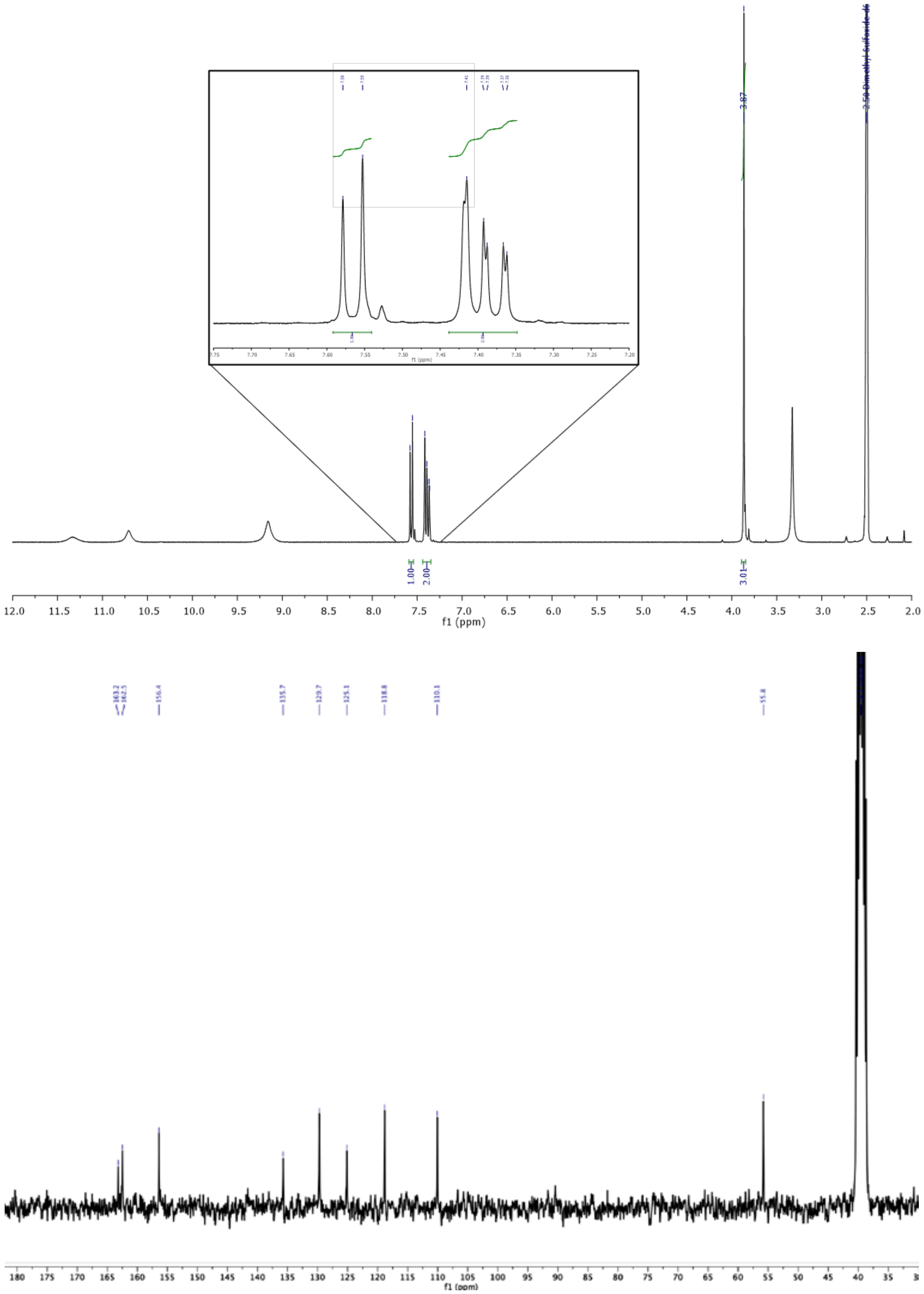

Figure S3. ^{1}H-NMR spectrum of compound *p*-H_4bdha-OCH_3 in DMSO-d_6 (top) and ^{13}C-NMR spectrum of compound *p*-H_4bdha-OCH_3 in DMSO-d_6 (bottom).

Preparation of 1,4-benzo-2-hydroxy-dihydroxamic acid (*p*-H_4bdha-OH)

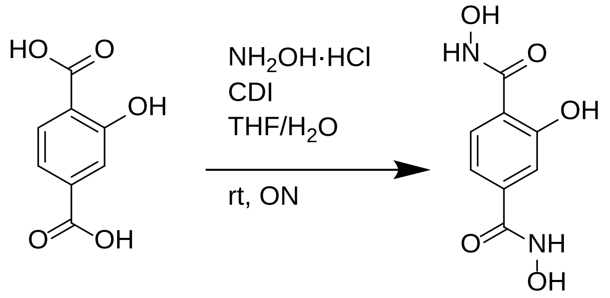

Scheme S4. Synthesis of *p*-H_4bdha-OH ligand.

1,1'-Carbonyldiimidazole (CDI) (1.95 g, 12 mmol) was dissolved in 75 mL of tetrahydrofuran (THF). Then, The solution was added to a suspension of 2-hydroxyterephthalic acid (0.73 g, 4 mmol) in methanol (25 mL). Later, a hydroxylamine hydrochloride (1.11 g, 16 mmol) solution (10 mL of deionized water) was added dropwise. The resulting mixture was stirred for 18 hours at room temperature and subsequently acidified to pH 6 with a solution of 30 % acetic acid. The yellow solid was filtered and washed with a mixture of acetone/deionized H_2O (4:1). The desired solid *p*-H_4bdha-NH_2 was dried under reduced pressure overnight (76 % yield). ^{1}H-NMR (300 MHz, DMSO-d_6) δ: 11.01 (brs), δ 8.96 (d, J = 18.1 Hz, 2H), 7.33 (d, J = 8.1 Hz, 1H), 7.10 (s, 1H), 6.79 (d, J = 8.1 Hz, 1H).^{13}C-NMR (75 MHz, DMSO-d_6) δ: 164.1 (C), 149.1 (C), 135.7 (C), 127.6 (CH), 115.0 (CH), 112.6 (CH).

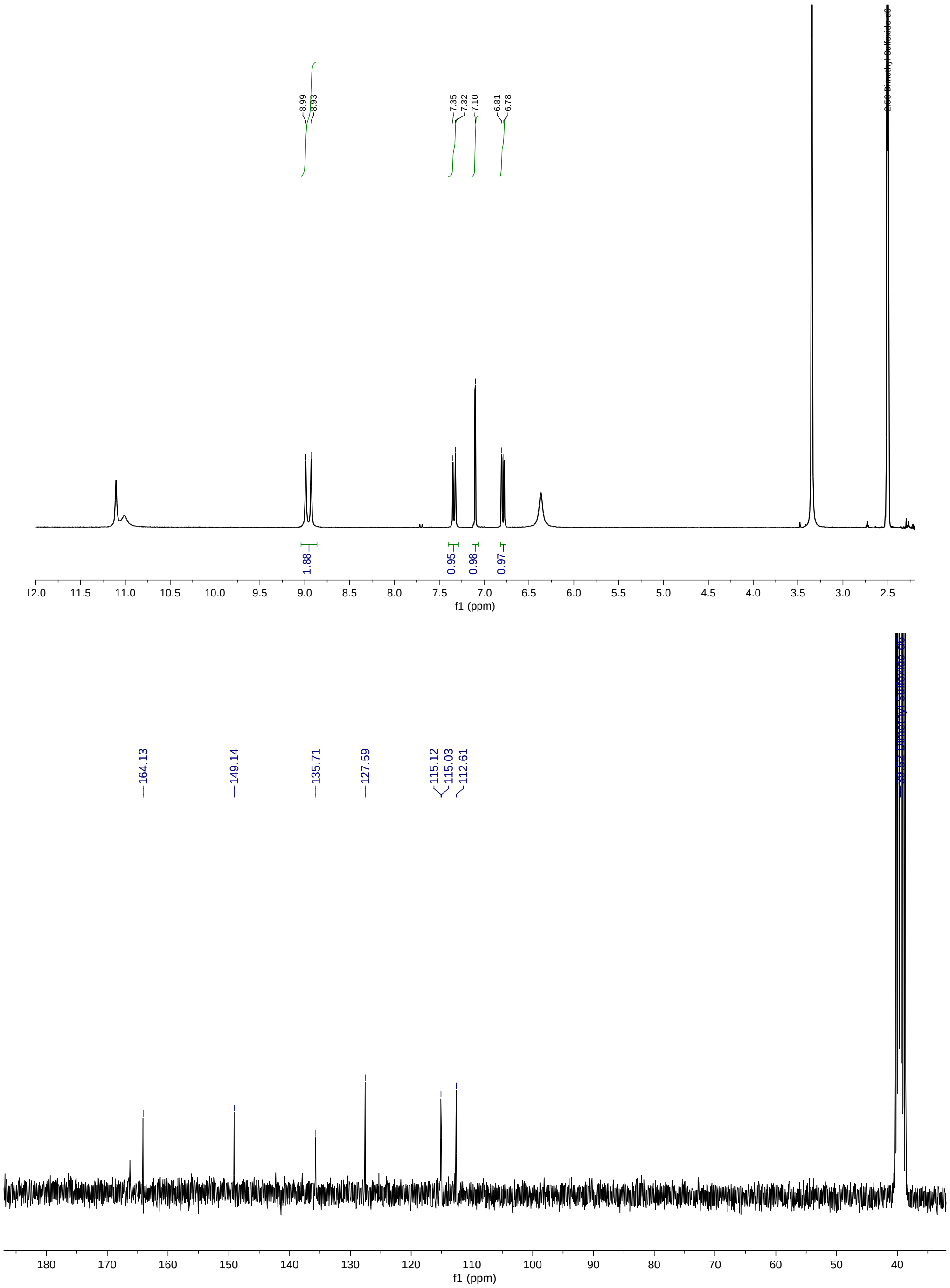

Figure S4. ^{1}H-NMR spectrum of compound *p*-H_4bdha-OH in DMSO-d_6 (top) and ^{13}C-NMR spectrum of compound *p*-H_4bdha-OH in DMSO-d_6 (bottom).

S2.2. Synthesis of cMUV-11

p-H_4bdha (106 mg; 0.54 mmol) and benzoic acid (165 mg, 1.36 mmol) were suspended in 36 mL of dry *N,N*-dimethylformamide (DMF) in a 50 mL Teflon vial. The mixture was sonicated for 5 minutes. Then, titanium (IV) isopropoxide (55 µL, 0.18 mmol) was added to the suspension. The vial was sealed and heated in a pre-heat oven at 100 °C for 18 hours.

After natural cooling down to room temperature, this results in the formation of octahedral deep red crystals that were isolated by centrifugation and washed with 60 mL of DMF (3x20 mL). The product was next soaked in fresh aliquots of acetone for 6 days. (Yield ca. 45 % based on Ti). Synthetic yield was calculated by using the molecular formula of desolvated material ([Ti_8(*p*-H_2bdha)$_8$(*p*-bdha)$_4$]). To minimize the presence of solvent in the solid, fresh crystals were washed with DMF and exchanged for 6 days with fresh acetone. Next, sample was heated in a vacuum oven for 24 h at 130 ºC. Elemental analysis for [Ti_8(*p*-H_2bdha)$_8$(*p*-bdha)$_4$]·$(H_2O)_{6.15}$·$(C_3H_7NO)_{2.6}$: Calc. C (41.48), H (3.71), N (12.4); found: C (41.92), H (3.61), N (11.96).

S2.3. Synthesis of cMUV-11-NH_2 & cMUV-11-OCH_3

By using the synthetic conditions optimized for cMUV-11 as starting point, we ran some reactions with *p*-H_4bdha-NH_2 and *p*-H_4bdha-OCH_3 as linkers to produce functionalized cMUV-11-X cages. These experiments also included systematic variations of the modulator with variable number of equivalents of benzoic acid, acetic acid or trifluoroacetic acid, by using DMF or mixtures of DMF and *N*-methyl-2-pyrrolidone (NMP) as solvent. The reaction time was also varied from 18 to 48 h. These experiments, that involved near to 50 reactions, were unsuccessful and did not allow for the formation of functionalized cMUV-11-X crystalline phases.

Hence, we opted for synthesising mixed-linker cMUV-11-NH_2 and -OCH_3 cages by following the same protocol used for cMUV-11 but using binary combinations of *p*-H_4bdha with *p*-H_4bdha-NH_2 or *p*-H_4bdha-OCH_3 at variable molar ratios ranging from 10 to 100%. We used a FLEX SHAKE high-throughput workstation from Chemspeed© for the automated dosing of solids to ensure maximum reproducibility. The vials were sonicated for 15 minutes to completely solubilize both linkers before placing in a pre-heat oven (100 °C) during 18h. After cooling down to room temperature, the deep red octahedral crystals were isolated by centrifugation and washed with DMF. The resultant crystals were soaked in DMF for further solvent exchange. **Table S1** summarizes the details for the synthesis of cMUV-11-X % cages.

Table S1. Summary of the synthetic conditions for mixed-linker cMUV-11-$X_{\%}$ cages.

% *p*-H_4bdha-X molar ratio	*p*-H_4bdha (mmol, mg)	*p*-H_4bdha-NH_2 (mmol, mg)	*p*-H_4bdha-OCH_3 (mmol, mg)
10	0.05, 95.4	0.49, 11.5	0.49, 12.2
20	0.43, 84.8	0.11, 23.0	0.11, 24.4
30	0.38, 74.0	0.16, 34.0	0.16, 36.6
40	0.324, 63.6	0.22, 42.0	0.22, 49.0
50	0.27, 53.0	0.27, 57.0	0.27, 61.7
60	0.22, 42.0	0.32, 69.0	0.32, 73.3
70	0.16, 32.0	0.38, 80.0	0.38, 86.0
80	0.11, 21.2	0.43, 91.2	0.43, 98.9
90	0.05, 10.6	0.49, 103.0	0.49, 111.3

It should be noted that in case of cMUV-11-NH_2, the samples prepared for higher *p*-H_4bdha-NH_2 loadings (70-90%) were obtained with remarkable low yields (around 10 %). As result, samples cMUV-11-NH_2 70, 80 and 90% could not be analysed with gas sorption experiments. In case of cMUV-11-OCH_3, *p*-H_4bdha-OCH_3 loadings over 70 % did not produce any solid. The maximum loading compatible with solid formation was 70%. However, the amount of material isolated was quite small and prevented gas adsorption analysis for cMUV-11-OCH_3 70%. As in the case of cMUV-11, synthetic yields were calculated by using the molecular formula (proposed by ^{1}H-NMR analysis) of desolvated materials in all cases, by following the same procedure. Results are summarized in the **Table S2**.

^{1}H-NMR Analysis. Incorporation ratio.

The percentage of functionalization was evaluated by ^{1}H-NMR analysis. For that, 10 mg of cMUV-11-X % were suspended in 0.5 mL of DMSO-d_6. After that, the sample were digested by addition of two drops of D_2SO_4-d_2 (96-98 wt. %). The mixture was stirred for 5 minutes at 80 °C. Then, the resulting clear solution was transferred to an NMR tube and ^{1}H-NMR was recorded. The ^{1}H-NMR analysis and peak integration was carried out by using the software MestReNova (version 6.0.2-5475). The characteristic aromatic peaks for each linker were selected by using the '*peak by peak'* option followed by integration with '*Autodetect Regions'* and '*Peaks*' as the calculation method, that does not provide a standard deviation for the corresponding integrals. The ratio of *p*-H_4bdha, *p*-H_4bdha-NH_2 and *p*-H_4bdha-OCH_3 incorporated to the material was calculated from the integration value of the aromatic signal for each ligand following the next equation:

$$\%\,\boldsymbol{p}-\mathbf{H4bdha} = \frac{\dfrac{\textit{Integral of the } \boldsymbol{p}-\mathbf{H4bdha}}{\text{Number of analyte protons of } \boldsymbol{p}-\mathbf{H4bdha}}}{\dfrac{\textit{Integral of the } \boldsymbol{p}-\mathbf{H4bdha}}{\text{Number of analyte protons of } \boldsymbol{p}-\mathbf{H4bdha}} + \dfrac{\textit{Integral of the } \boldsymbol{p}-\mathbf{H4bdha}-\mathbf{X}}{\text{Number of analyte protons of } \boldsymbol{p}-\mathbf{H4bdha}-\mathbf{X}}} \times 100$$

$$\%\ \mathbf{of}\ \boldsymbol{functionalization} = 100 - \ \%\,\boldsymbol{p}-\mathbf{H4bdha}$$

An example of the calculation of the incorporation ratio for cMUV-NH_2 85% is shown below. The percentage of incorporation is summarized in **Table S2**.

$$\%\,\boldsymbol{p}-\mathbf{H4bdha} = \frac{\frac{0.72}{4}}{\frac{0.72}{4} + \frac{1}{1}} \times 100 = 15\%$$

$$\%\ \mathbf{of}\ \boldsymbol{functionalization} = 100 - \ 15 = 85\%$$

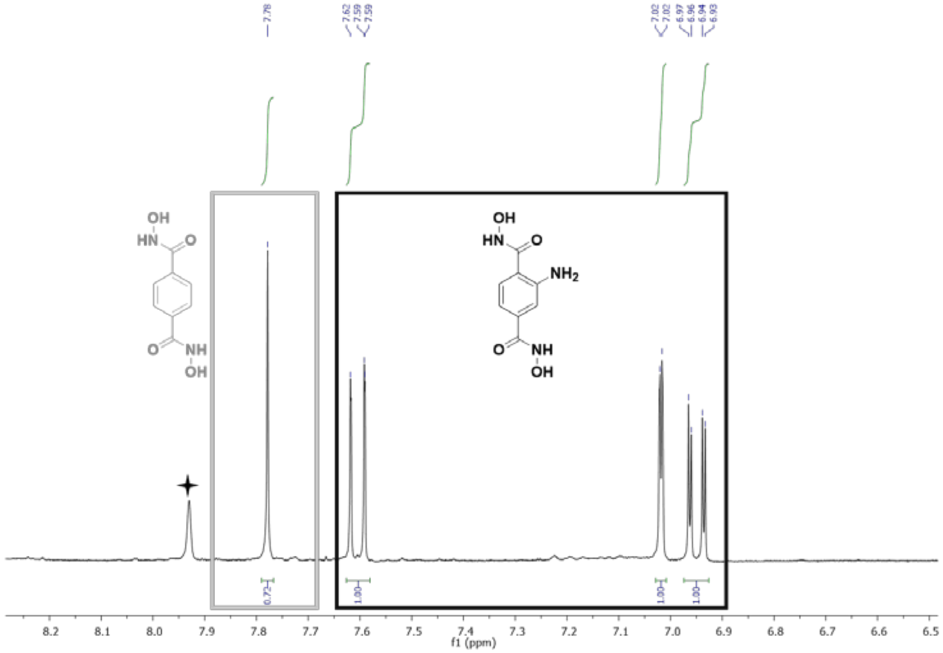

Figure S5. ^{1}H-NMR of cMUV-NH_2 85% sample after digestion with D_2SO_4-*d₂*. The characteristic region of each ligand is framed in grey and black squares. The residual DMF signal is indicated with a black star.

a

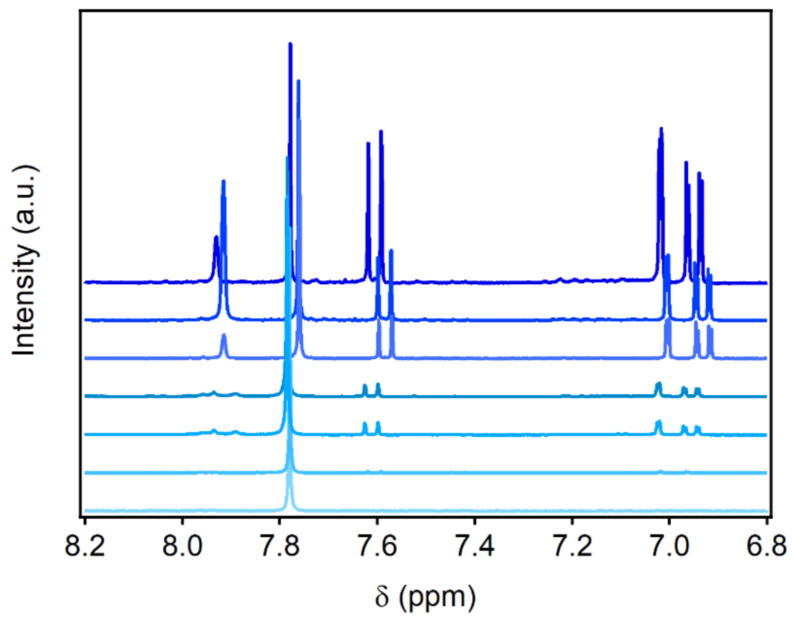

b

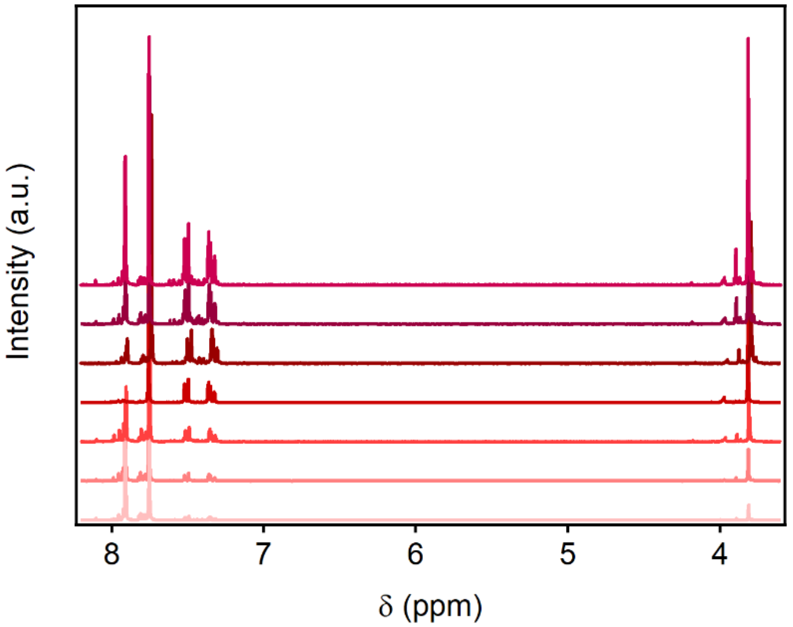

Figure S6. a) ^{1}H-NMR spectra of digested cMUV-NH_2 % samples. b) ^{1}H-NMR spectra of digested cMUV-OCH_3 % samples. The slight displacement of the signals is due the amount of D_2SO_4-*d_2* used in the digestion process. The signal at 7.9 ppm corresponds to residual DMF.

Table S2. Summary of the percentage of functionalization (calculated by ^{1}H-NMR analysis) and yields for cMUV-11-X$_{\%}$ cages.

% Functional group added	%-NH_2 incorporated	Yield (%) cMUV-NH_2	% -OCH_3 incorporated	Yield (%) cMUV-OCH_3
10	Negligible	---	11.4	48
20	Negligible	---	21.3	51
30	4.7	45	33.2	53
40	8.1	40	44.1	45
50	28.6	41	53.2	46
60	48.9	33	60.7	42
70	59.0	27	69.3	37
80	70.4	12	No solid formed	---
90	84.6	8	No solid formed	---

S3. cMUV-11 structures. Single-Crystal X-ray Diffraction Analyses

X-ray diffraction data on a single crystal were collected on a Bruker D8 Advance diffractometer with Photon III using CuKα radiation (λ = 1.54184 Å) at 100(1)K by using APEX3[4] software. Images were converted to Esperanto format in order to use CrysAlisPro[5] for data reduction, scaling and absorption correction for cMUV-11 and cMUV-11-NH_2. In the case of cMUV-11-OCH_3, we used APEX3, SAINT-Plus and SADABS[4]. The structure was solved applying the dual-space algorithm implemented in SHELXT program[6]. Fourier recycling and least-squares refinement were used for the model completion with SHELXL-2018[6]. All non-hydrogen atoms have been refined anisotropically, and all hydrogen atoms have been placed in geometrically suitable positions and refined riding with isotropic thermal parameter related to the equivalent isotropic thermal parameter of the parent atom. The geometrical analysis of interactions in the structure was performed with the Olex2 program[7]. The hydrogen atoms were geometrically positioned with C-H = 0.93Å and Uiso(H) = 1.2 Ueq(C). Crystal data, collection procedures and refinement results are summarized in **Table S3**. In both cases, the organic linker presents a certain degree of disorder respect to the -NH2 and -OCH3 groups linked to the benzene ring. Also, as usual for this type of compounds, the structure contains a large amount of disordered solvent (DMF molecules) in the voids that was impossible to model correctly. Thus, the squeeze procedure was applied at the end of the refinement process in all cases. Crystallographic data for the structures reported in this contribution have been deposited with the Cambridge Crystallographic Data Centre and as supplementary publication files. Copies of the data can be obtained free of charge on application to the CCDC, Cambridge, U.K. (http://www.ccdc.cam.ac.uk/) or available as supplementary files.

Table S3. Crystallographic data of cMUV-11 derivatives.

Compound	cMUV-11	cMUV-11-NH$_2$	cMUV-11-OCH$_3$
Identification code	CCDC2106823	CCDC2106824	CCDC2106822
Empirical Formula	$C_{25.5}H_{39.5}N_{7.5}O_{10.5}Ti$	$C_{19.5}H_{25.65}N_{6.15}O_{8.5}Ti$	$C_{23.19}H_{33.63}N_{6.5}O_{10.19}Ti$
Formula Weight	667.04	530.11	614.41
T/K	100(1)	100(1)	100(1)
Crystal System	Tetragonal	Tetragonal	Tetragonal
Space Group	I4/m	*I4/m*	*I4/m*
a/Å	24.0640(4)	24.0023(3)	23.9384(15)
b/Å	24.0640(4)	24.0023(3)	23.9384(15)
c/Å	24.6958(11)	24.7860(14)	24.913(2)
α/˚	90	90	90
β/˚	90	90	90
γ/˚	90	90	90
V/Å^3	14300.7(8)	14279.5(9)	14276(2)
Z	16	16	16
$\rho_{calc.}$/ g cm^{-3}	1.239	0.986	1.143
μ/mm^{-1}	2.541	2.389	2.491
Crystal size/mm^3	0.10×0.09×0.08	0.11×0.08×0.07	0.10×0.08×0.08
Radiation	Cu K$_\alpha$	Cu K$_\alpha$	Cu K$_\alpha$
2ϑ range/ º	5.128 - 130.174	8.836 - 130.062	5.12 - 118.898
Reflections collected	37676	32576	21207
Independent reflections	6239	6185	4861
Refl's with I > 2(I)	4518	3808	2728
Parameters/ restraints	200/0	218/0	238/8
Largest Peak / hole e·Å^3	1.23/ -0.46	1.21/ -0.36	0.47/-0.28
Goodness-of-fit on F^2	1.101	1.002	1.039
Final R indexes [I≥ 2σ (I)]	wR_2=0.2635;R_1=0.0927	wR_2=0.2472;R_1=0.0850	wR_2=0.2215;R_1=0.0800
Final R indexes [all data]	wR_2=0.2839;R_1=0.1061	wR_2=0.2715;R_1=0.1051	wR_2=0.2530;R_1=0.1174

Table S4. Hydrogen bonds[8] in cMUV-11-X (X = H, NH_2 and OCH_3) cages.

cMUV-11

Donor-H…Acceptor	d(D-H) (Å)	d(H-A) (Å)	d(D-A) (Å)	D-H-A (°)
N2-H2…O3i	0.88	1.77	2.621(4)	164
N2-H2…N3^{i}	0.88	2.51	3.245(5)	142
C4-H4…O6	0.95	2.47	2.796(5)	100
C12-H12…O4	0.95	2.46	2.789(6)	100

(i) 1/2-x, 3/2-y, 3/2-z

cMUV-11-NH_2

Donor-H…Acceptor	d(D-H) (Å)	d(H-A) (Å)	d(D-A) (Å)	D-H-A (°)
N2-H2…O3i	0.88	1.76	2.607(4)	161
N1-H2…N4	0.88	2.11	2.753(15)	129
N2-H2…N3i	0.88	2.49	3.228(5)	142
C3-H3A…O2	0.95	2.44	2.768(5)	100
C12-H12…O4	0.95	2.47	2.798(6)	100

(i) 3/2-x, 3/2-y, 1/2-z

cMUV-11-OCH_3

Donor-H…Acceptor	d(D-H) (Å)	d(H-A) (Å)	d(D-A) (Å)	D-H-A (°)
N2-H2…O6	0.88	1.77	2.600(6)	156
N1-H1…O8	0.88	2.14	2.739(15)	125
C4-H4…O3	0.95	2.44	2.765(7)	100
C12-H12…O5	0.95	2.46	2.787(9)	100

(i) 1/2-x, 1/2-y, 1/2-z

Table S5. Porosity of pristine and functionalized cMUV-11 cages as estimated from Olex2 CalcVoid/CalcSolv analysis with a 0.2 Å resolution.

	Radius largest spherical void [Å]	Structure volume		Cell vol. [Å^3]	Penetration sphere radius [Å]			Solvent accessible	
		Vol. [Å^3]	[%]		a	b	c	Vol. [Å^3]	[%]
cMUV-11	7.0	3586.4	25.1	14300.7	3.6	3.6	3.6	10126.4	70.8
cMUV-11-NH_2	7.2	3867.6	27.1	14279.5	3.2	3.2	3.8	9720.6	68.1
cMUV-11-OCH_3	7.2	4107.7	28.8	14276.3	2.6	2.6	3.8	9478.8	66.4

CalcVoid run using –p –r=0.1 & CalcSolv solvent_radius of 1.80 Å & shrink truncation_radius of 1.80 Å. Van der Waals radii: C, 1.77; H, 1.20; O, 1.52; and Ti, 2.00 Å.

S4. Chemical characterization

S4.1. cMUV-11

Scanning Electron Microscopy (SEM)

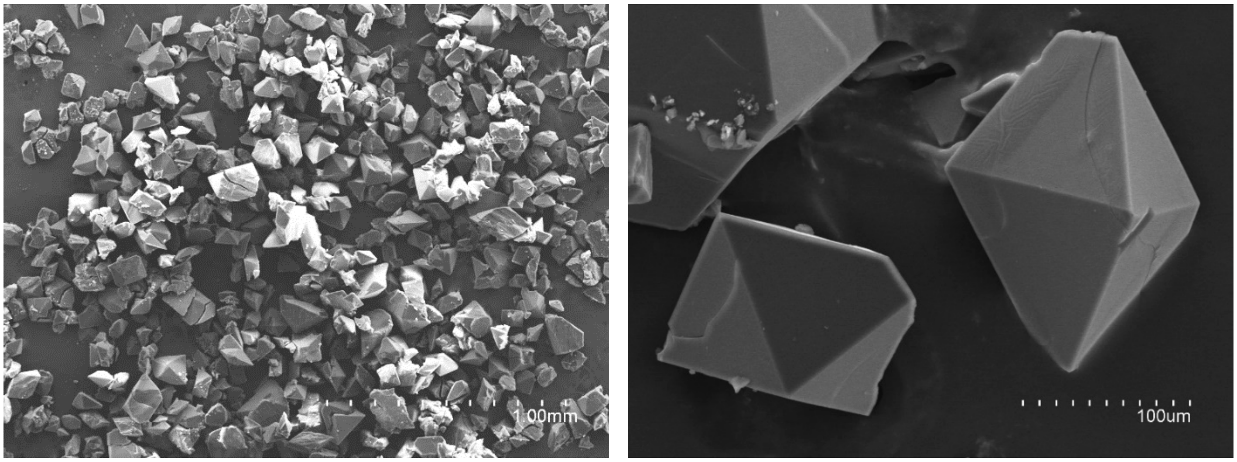

Figure S7. Scanning Electron Microscopy (SEM) images of crystal of cMUV-11. Lower magnification confirms the formation the homogeneity of the sample. Higher magnification shows the particle size is between 50-100 µm.

Thermogravimetric Analysis (TGA)

cMUV-11 shows a first weight loss centred at 210 °C due the early decomposition of the *p*-H_4bdha units. This agrees well with the profile for the thermal decomposition of the free linker (grey line). This decomposition profile is quite similar to the extended framework MUV-11 based on the same connector.[2] The total decomposition of the MOP takes place above 400 °C to form 21.10 % of TiO_2 (Calc.: 23.62%).

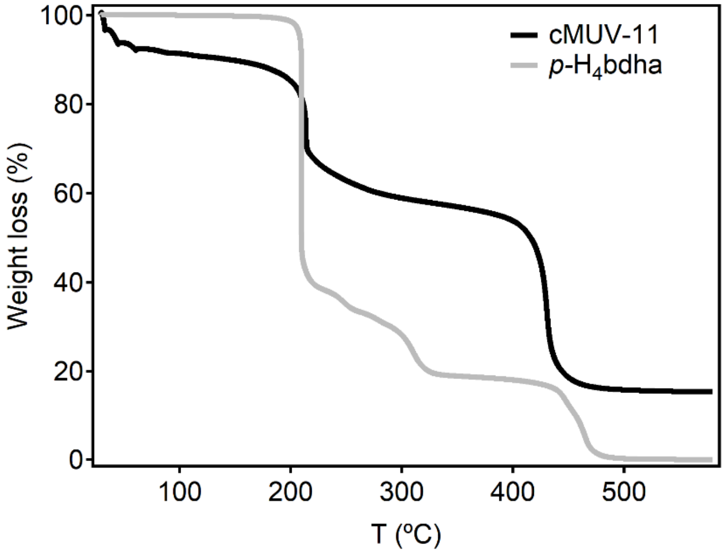

Figure S8. Thermogravimetric analysis of cMUV-11 (black line) and the organic ligand *p*-H_4bdha (grey line).

Powder X-Ray Diffraction (PXRD)

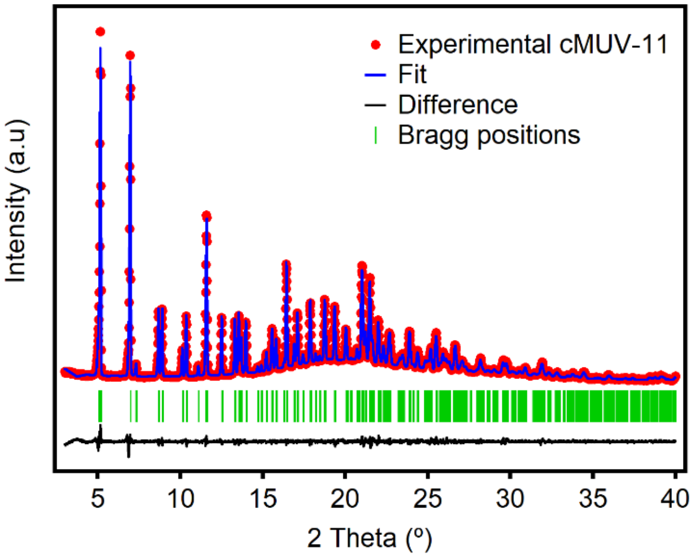

Figure S9. Experimental (red dots), calculated (blue line), difference plot [(Iobs−Icalc)] (black line) and Bragg positions (green ticks) for the refinement of the experimental diffraction data of cMUV-11 soaked in DMF collected at room temperature by using the Le Bail method using the single-crystal structural model as starting parameters. Tetragonal, I 4/m (87); a = b = 24.1052 c = 25.3229 Å; α = β = γ = 90°; V = 14714 $Å^3$; Re = 2.45 %, Rp = 2.99 %, Rwp = 4.22 %, GoF = 1.72.

S4.2. Solubility test of cMUV-11

We run a systematic study of the solubility of the as-made crystals in simple and binary combinations of solvents that cover a broad range of polarities and account for the most representative conditions reported for solubilizing MOPs:

Table S6. Representative solvents or mixtures of solvents used for solubilizing MOPs extracted from the literature. In a typical experiment, 2 mg of freshly made crystals of cMUV-11 were dispersed in 5 mL of the corresponding solvent and heated at 60º C for 24 hours in a 10 mL capped vial.

Solvent	Reference	Polarity	Solubility of cMUV-11
Water	9	1	Not soluble
Methanol	9	0.762	Partial solubility(<<0.2 $mg \cdot mL^{-1}$)
Dimethylsulfoxide (DMSO)	9	0.444	Partial solubility (0.2 $mg \cdot mL^{-1}$)
Dimethylacetamida (DMA)	9,10	0.398	Partial solubility (0.2 $mg \cdot mL^{-1}$)
Dimethylformamide (DMF)	9	0.386	Partial solubility (<0.2 $mg \cdot mL^{-1}$)
DMF/water (3:1 v/v%)	11	----	Partial solubility
Acetonitrile	9	0.355	Not soluble
Acetone	9	0.355	Not soluble
Tetrahydrofuran (THF)	12	0.207	Not soluble

Dispersion in CH_3OH, DMSO, DMF, DMF/water (3:1), DEF or DMA resulted in the formation of orange/yellowish solutions of variable colour intensity that coexisted with the presence of crystals in suspension. We initially ascribed this to the partial solubilization of the cage in these conditions. For further probe, we attempted to solubilize cMUV-11 in the same conditions by using DMSO-d_6 for analysis of the solution with ^{1}H-NMR after filtering the suspension. Comparison of the spectra of the free linker and cMUV-11 suggests that the cage is present in solution based on the shift of the aromatic proton signal of the linker from 7.80 to 7.90 ppm likely due to the effect of Ti(IV) coordination. We decided to confirm this point further by MALDI TOF TOF-MS of the supernatants. It is worth noting that we could not use to milder ionization conditions as ESI because the quadrupole detector is limited to 1500-2000 m/z and would not be compatible with the m/z ratio of the unfragmented cage. As shown in **Figure S10, *bottom***, none of the spectra collected are consistent with the presence of the signals that would correspond to a clean ionization of the $[cage]^{n+}$ (n=1-8) at m/z values of 2704.9, 1352.4, 901.6, 676.2, 541.0, 450.8, 386.4 or 338.1, and the complex fragmentation patterns observed suggest instead a partial fragmentation in the conditions of the experiment possibly due to the aggressive ionization conditions.

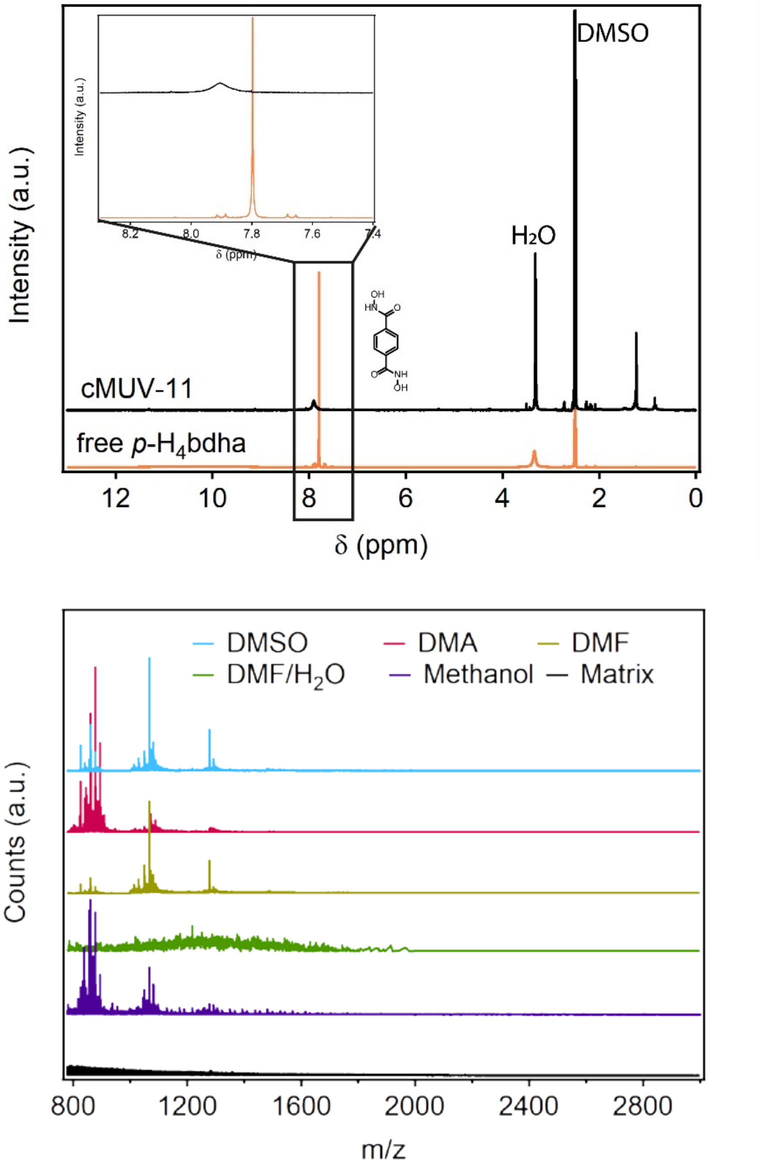

Figure S10. *Top*) ^{1}H-NMR spectra of the free linker and cMUV-11 in DMSO-d_6. Residual solvent peak of DMSO-d_6 at 2.50 ppm and of D_2O at 3.33 ppm. *Bottom*) MALDI TOF TOF-MS spectra the orange/yellowish supernatants that result from partial solubilization of cMUV-11 crystals in the different solvents explored.

We have also tested the solubility of the cage in deuterated methanol (CD_3OD) in the same conditions. The ^{1}H-NMR of the supernatant suggests the presence of intact cages based on the same shift of the aromatic proton compared to the free linker (**Figure S11, *top***). The addition of tetrabutylammonium hydroxide (TBA(OH)), for facilitating the deprotonation of the single deprotonated chelates, resulted in the complete dissolution of the crystals for a transparent yellow solution. The NMR spectrum displays two signals that suggest the presence of the cage but also its possible degradation in these conditions. We also collected MALDI TOF TOF-MS spectra for both solutions. As described above, this ionization technique appears to be too aggressive and results in complex fragmentation patterns that cannot be assigned to cMUV-11 in any of its possible charge states.

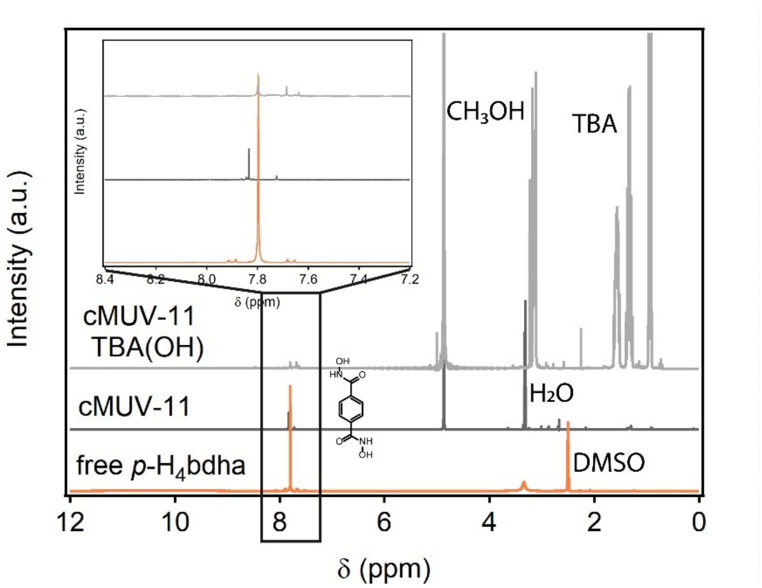

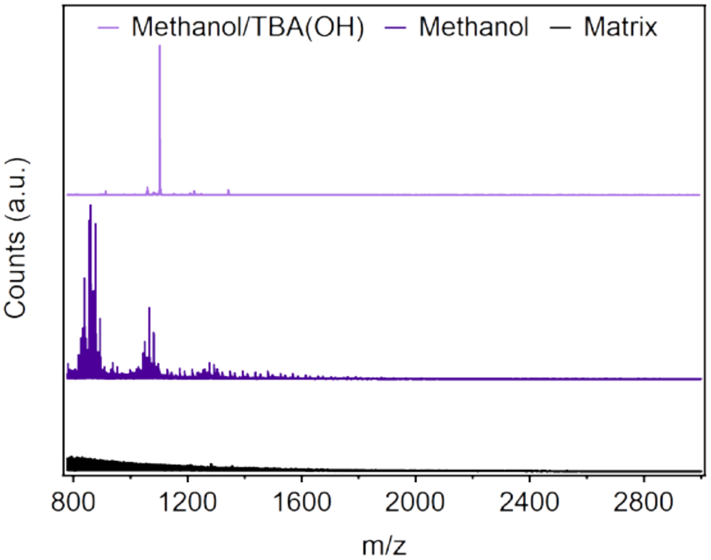

Figure S11. *Top*) ^{1}H-NMR spectra of the linker (DMSO-d_6), cMUV-11 dissolved in CD_3OD-d_4 and in a mixture of CD_3OD-d_4TBA(OH) (9:1). Residual solvent peaks of DMSO-d_6 (2.50), CD_3OD-d_4 (4.87) and D_2O (3.33 ppm). *Bottom*) MALDI TOF TOF-MS spectra of the orange/yellowish supernatants that result from partial solubilization of cMUV-11 crystals in different solvents. b) of resultant methanol and methanol/TBA(OH) supernatants.

S4.3. cMUV-11-NH_2 & cMUV-11-OCH_3

Scanning Electron Microscopy (SEM)

cMUV-11-NH_2 50% and cMUV-11-OCH_3 50% were selected to confirm the purity of the mixed-linker cages. The appearance of the samples (morphology and crystal size) is not modified either by the functional group or the percentage of loading.

cMUV-11-NH_2 50 %

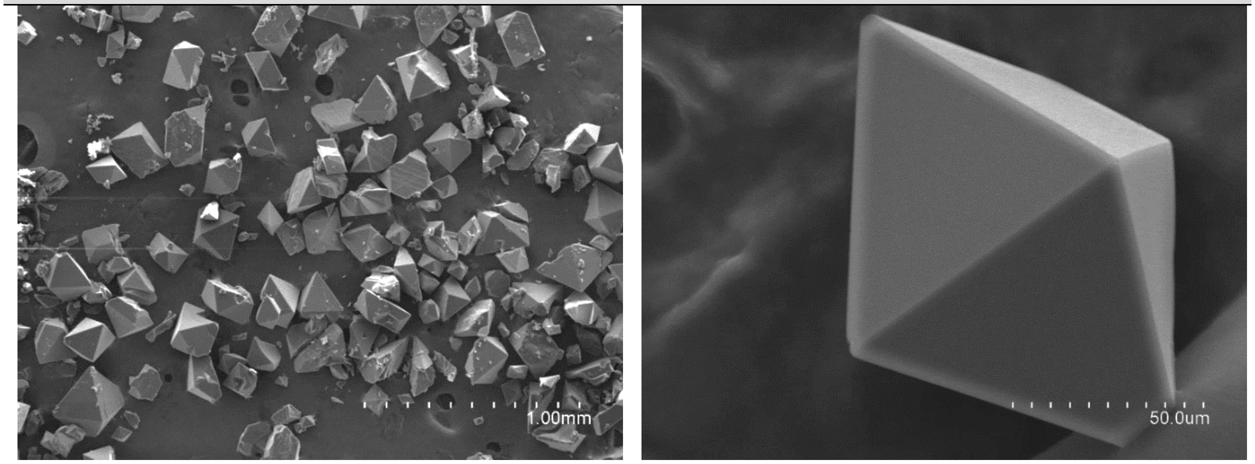

cMUV-11-OCH_3 50 %

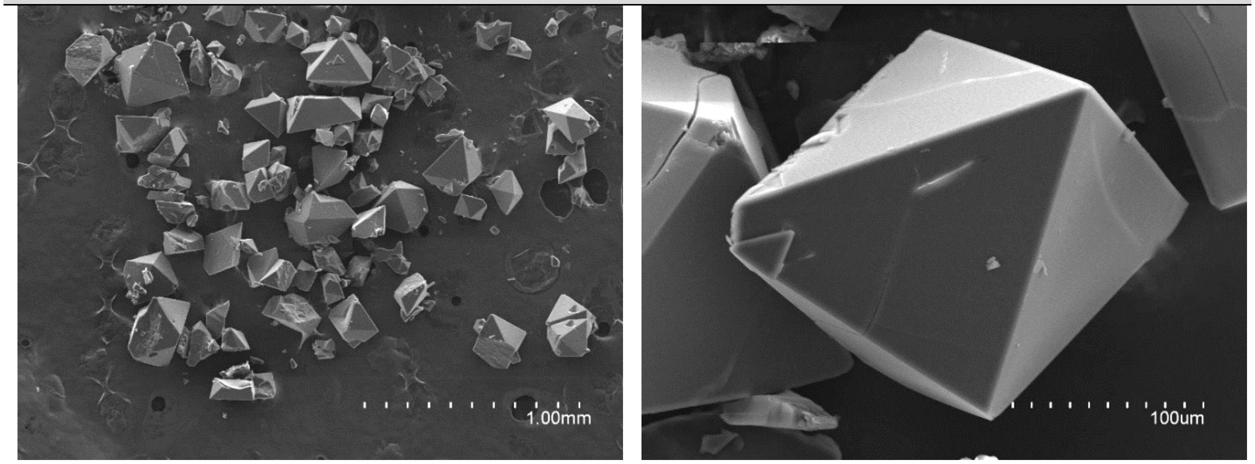

Figure S12. Scanning Electron Microscopy (SEM) images of crystal of cMUV-11-NH_2 (up) and cMUV-11-OCH_3 (down). Lower magnification confirms the formation the homogeneity of the samples. Higher magnification shows the particle size is around 50-100 µm.

Thermogravimetric Analysis (TGA)

The thermal decomposition profiles of the functionalized cages agree well with cMUV-11 and only display a minimum shift in the thermal stability to higher temperatures.

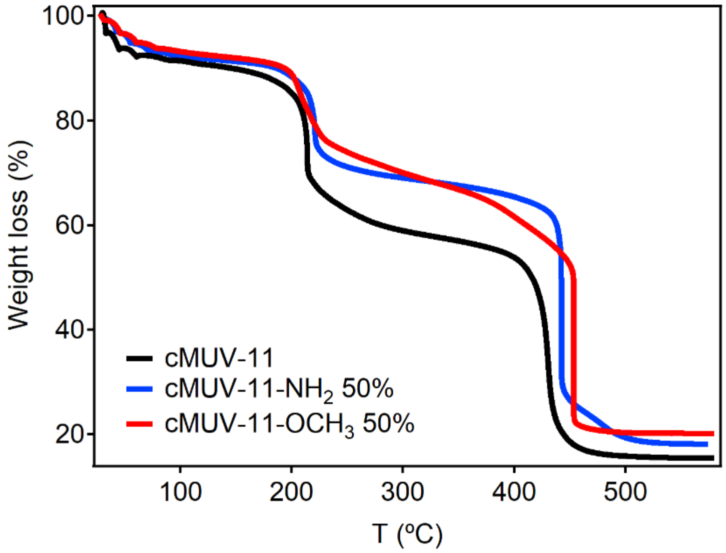

Figure S13. Thermogravimetric analysis of cMUV-11 (black line), cMUV-11-NH_2 50% (blue line) and cMUV-11-OCH_3 50% (red line).

Elemental analysis (EA)

<u>cMUV-11-NH_2 50%:</u> [Ti_8(*p*-H_2bdha)$_4$(*p*-H_2bdha-NH_2)$_4$(*p*-bdha)$_2$(*p*-bdha-NH_2)$_2$]·$(H_2O)_4$·$(C_3H_7NO)_4$: Calc. C (39.46), H (3.25), N (14.49); found: C (40.00), H (3.99), N (14.22).

<u>cMUV-11-OCH_3 50%:</u> [Ti_8(*p*-H_2bdha)$_4$(*p*-H_2bdha-OCH_3)$_4$ (*p*-bdha)$_2$(*p*-bdha-OCH_3)$_2$]·$(H_2O)_{3.75}$: Calc. C (41.19), H (3.57), N (11.19); found: C (41.58), H (3.69), N (10.80).

Powder X-Ray Diffraction (PXRD)

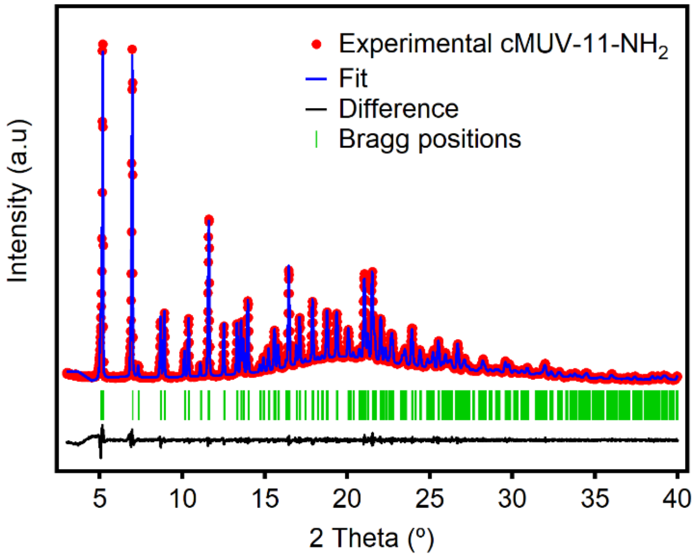

Figure S14. Experimental (red dots), calculated (blue line), difference plot [(Iobs−Icalc)] (black line) and Bragg positions (green ticks) for the refinement of experimental diffraction data of cMUV-11-NH_2 50% soaked in DMF collected at room temperature by using the Le Bail method using the single-crystal structural model as starting parameters. Tetragonal, I 4/m (87); a = b = 24.081c = 25.323Å; α = β = γ = 90°; V = 14684 Å^3; Re = 2.43 %, Rp = 3.07 %, Rwp = 4.88 %, GoF = 2.00.

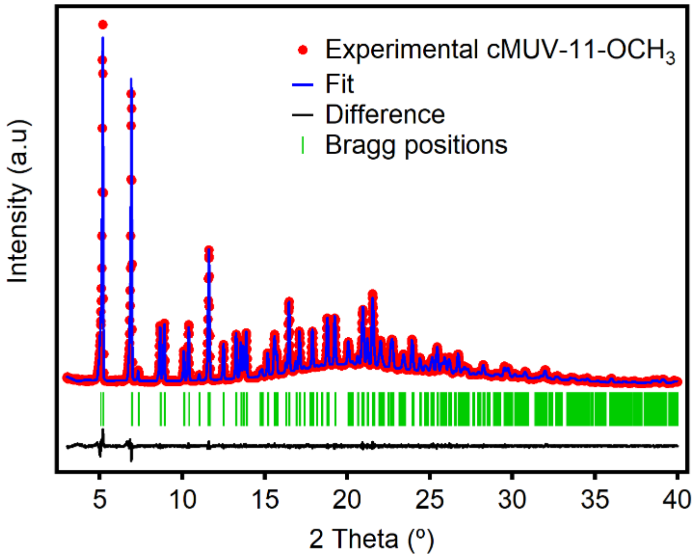

Figure S15. Experimental (red dots), calculated (blue line), difference plot [(Iobs−Icalc)] (black line) and Bragg positions (green ticks) for the refinement of experimental diffraction data of cMUV-11-OCH_3 50% soaked in DMF collected at room temperature by the LeBail method using the single-crystal structural model as starting parameters. Tetragonal, I 4/m (87); a = b = 24.0544 c = 25.5037 Å; α = β = γ = 90°; V = 14756 Å^3; Re = 2.52 %, Rp = 2.57 %, Rwp = 3.55 %, GoF = 1.41.

S5. Activation of cMUV-11 cages and N_2 adsorption

N_2 isotherms

cMUV-11

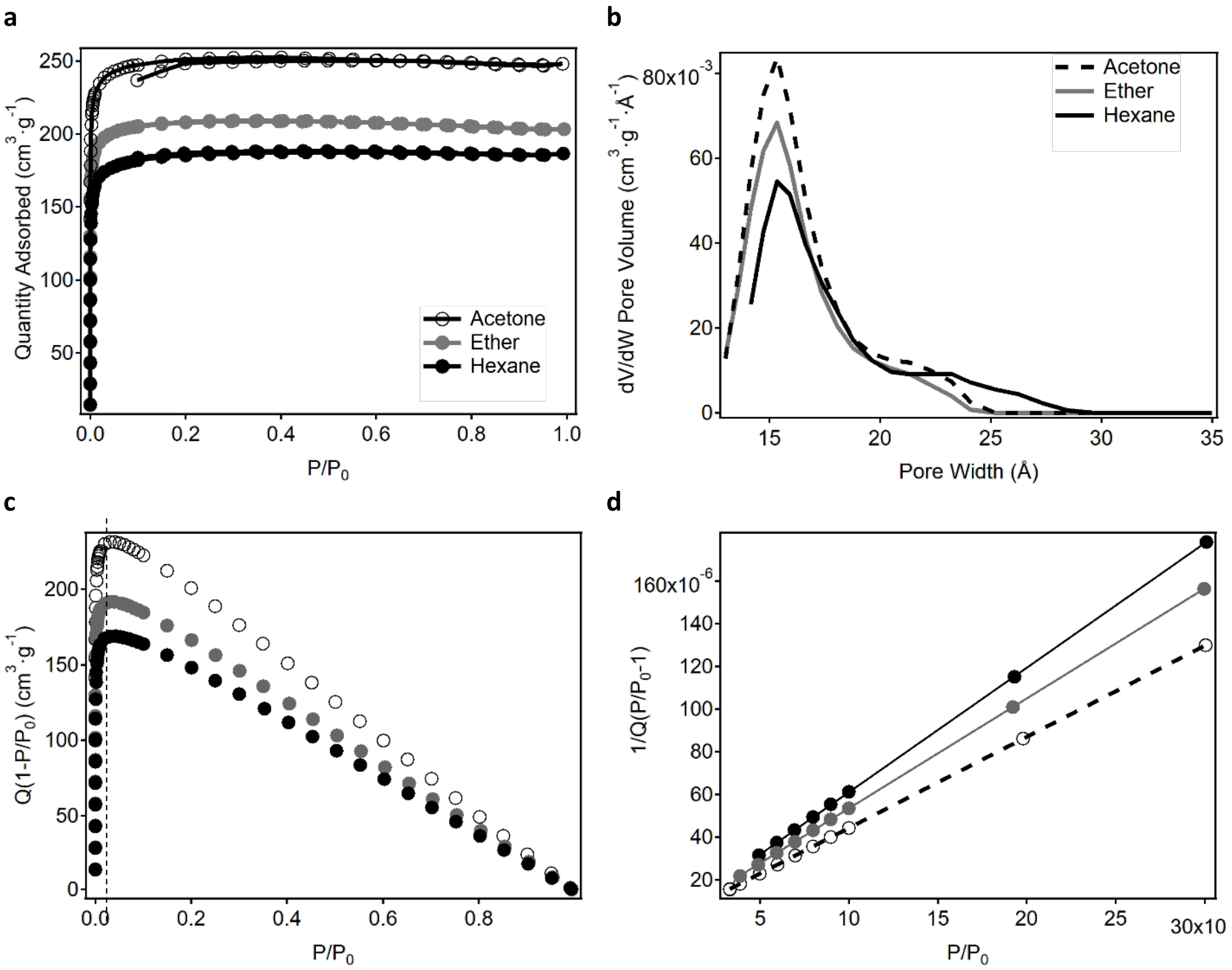

Figure S16. a) N_2 adsorption/desorption isotherms at 77 K. b) Pore size distribution calculated assuming a cylindrical pore model (N2@77-Carb Cyl Pores, MWNT, NLDFT) of cMUV-11 samples exchanged with different solvents. c) Rouquerol BET, the dashed line represents the limit of the applicability of the BET theory and d) Multi-Point BET analysis.

Table S7. Summary of the adsorption parameters for different solvent exchange for cMUV-11.

Solvent	BET surface area ($m^2 \cdot g^{-1}$)	V_p total at P/P_0 = 0.95 ($cm^3 \cdot g^{-1}$)
Acetone	1020	0.38
Ether	844	0.32
Hexane	747	0.29

Table S8. Summary of the main parameters calculated from the multi-point BET analysis of cMUV-11 exchanged samples.

Solvent	Slope ($g \cdot cm^{-3}$)	Intercept ($g \cdot cm^{-3}$)	Range P/P_0	R^2	C	V_m ($cm^3 \cdot g^{-1}$)	$1/(\sqrt{C}+1)$	P/P_0 at V_m
Acetone	0.004266 ± 0.000002	0.000002 ± 0.000000	0.0037-0.037	0.99999	2584.9644	234.3233	0.0193	0.0191
Ether	0.005153 ± 0.000004	0.000002 ± 0.000000	0.0037-0.037	0.99999	2655.8207	193.9797	0.0190	0.0168
Hexane	0.005825 ± 0.000004	0.000003 ± 0.000000	0.0037-0.037	0.99999	2013.1356	171.5913	0.0218	0.0203

cMUV-11-NH$_2$

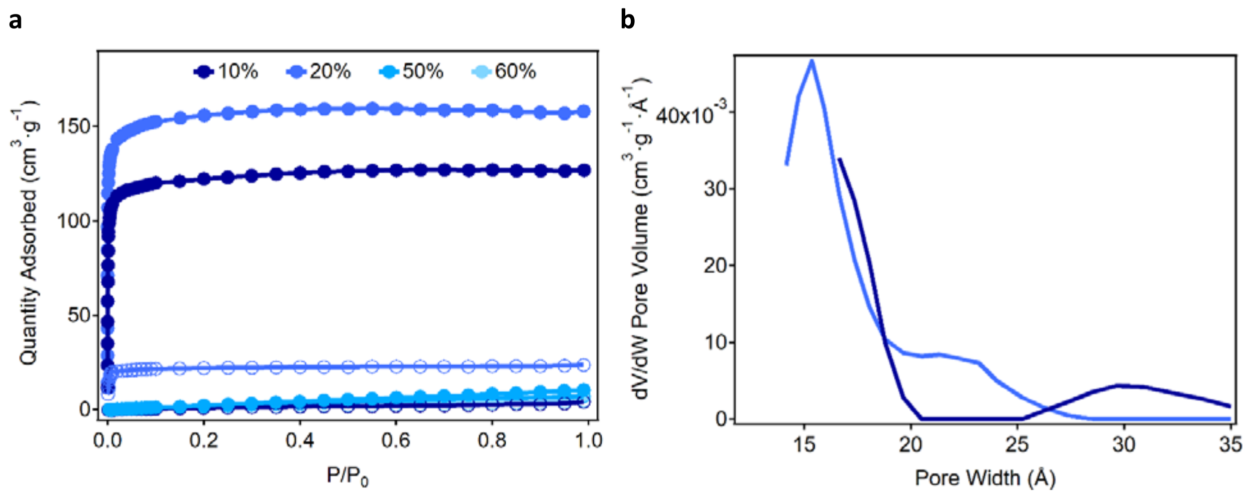

Figure S17. a) N_2 adsorption/desorption isotherm at 77 K and b) Pore size distribution calculated assuming a cylindrical pore model (N2@77-Carb Cyl Pores, MWNT, NLDFT) of cMUV-11-NH$_2$ % (X = 10, 20, 50 & 60%) exchanged with acetone (empty symbols) or hexane (filled symbols).

Table S9. Summary of the adsorption parameters for different solvent exchange for cMUV-11-NH_2 samples.

% NH_2 group	Solvent	BET surface area ($m^2 \cdot g^{-1}$)	V_p total at P/P_0 = 0.95 ($cm^3 \cdot g^{-1}$)
10	Acetone	---	---
10	Hexane	493	0.20
20	Acetone	98	0.04
20	Hexane	620	0.24
50	Acetone	84	0.03
50	Hexane	---	---
60	Acetone	---	---
60	Hexane	---	----

cMUV-11-OCH_3

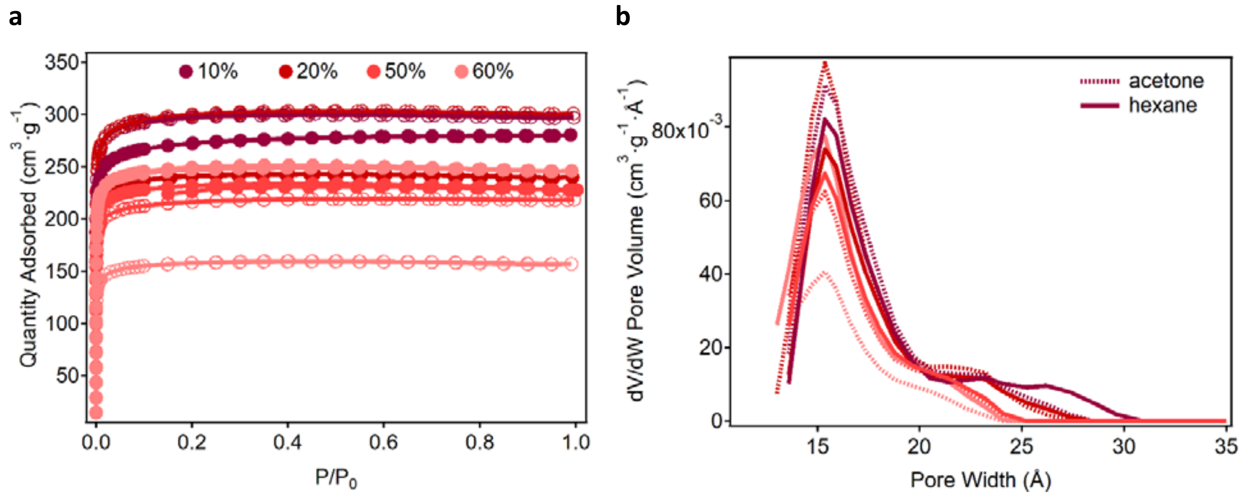

Figure S18. a) N_2 adsorption/desorption isotherm at 77 K of and b) Pore size distribution calculated assuming a cylindrical pore model (N2@77-Carb Cyl Pores, MWNT, NLDFT) of cMUV-11-OCH_3 % (X = 10, 20, 50 & 60%) exchanged with acetone (empty symbols) or hexane (filled symbols). b) The pore size distribution for exchanged cages is also centred at 15 Å. The dotted-line represents acetone exchange and solid-line hexane exchange.

Table S10. Summary of the adsorption parameters for different solvent exchange for cMUV-11-OCH_3 samples.

% OCH_3 group	Solvent	BET surface area ($m^2 \cdot g^{-1}$)	V_p total at P/P_0 = 0.95 ($cm^3 \cdot g^{-1}$)
10	Acetone	1206	0.46
10	Hexane	1098	0.43
20	Acetone	1215	0.47
20	Hexane	977	0.37
50	Acetone	875	0.34
50	Hexane	888	0.33
60	Acetone	636	0.24
60	Hexane	1009	0.38

Table S11. Comparison of experimental and theoretical textural properties of the cMUV-11-X family. 50% functionalization is selected as representative of the series.

Sample	SA_{BET} [$m^2 \cdot g^{-1}$]		Pore volume [$cm^3 \cdot g^{-1}$]	
	exp[a]	theor	exp[b]	Theor
cMUV-11	1020	3350	0.38	0.96
cMUV-11-NH_2-50%	84	3095	0.03	0.83
cMUV-11-OCH_3-50%	888	2535	0.33	0.72

[a]Specific surface area (SA) was calculated by multi-point Brunauer-Emmett-Teller (BET) method. [b]Total pore volume at P/P_0=0.96.

The theoretical accessible surface areas and probe-occupiable volumes of the cage variants (-H, -NH_2, -OCH_3) from their crystallographic structures were calculated by using the software package Zeo++ with a probe size representative of the kinetic diameter of N_2 (chan_radious=probe_ratious=1.82 and num_samples=100000).[13] The experimental values are significantly below the theoretical expectations confirming the structural sensitivity of cMUV-11 to solvent evacuation and the importance of retaining the crystalline arrangement for optimum porosity values. This is particularly important for cMUV-11-NH_2, for which the incorporation of polar -NH_2 groups induces more drastic structural changes upon solvent removal. This is consistent with the discussion in the main text and the postulated role of -X cage functions in affecting the structural response of the cage packing to solvent evacuation.

S6. Chemical and Structural Stability

ICP-MS measurements

The hydrolytic stability of the solids was evaluated by incubation in water at different pHs for 24 hours (10 mg·mL^{-1} concentration).

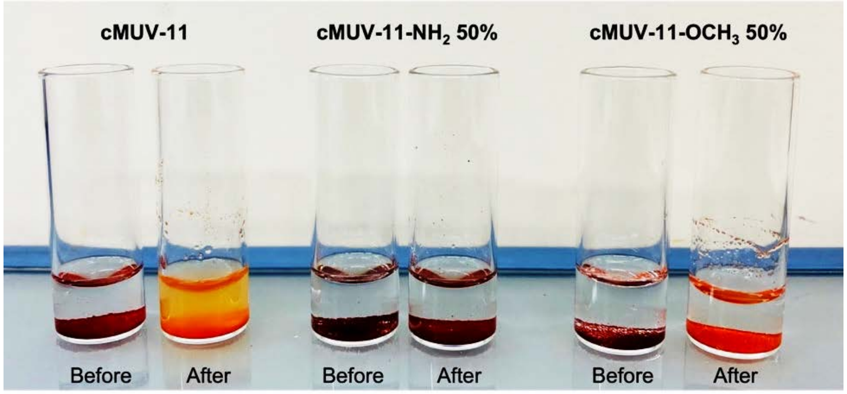

Figure S19. Picture of samples before and after chemical stability test.

Table S12. Values of titanium concentration (ppm) in solution of different standard Ti-MOFs after soaking in water pH = 7 for 24 hours compared with values reported in this work.

Material	Ti^{+4} in solution (ppm)
cMUV-11	4.04 ± 0.05
cMUV-11-NH_2 50%	0.29 ± 0.01
cMUV-11-OCH_3 50%	2.02 ± 0.04
MUV-11	0.02 ± 0.04
MIL-125 NH_2	0.83 ± 0.03
MIL-100 (Ti)	0.12 ± 0.01

To complete the hydrolytical stability study, crystals of cMUV-11 variants were incubated in water at pH = 3, 5, 7, 9 and 11 for 24 hours. The resultant supernatants were also analysed by ICP-MS.

As shown in **Figure S20**, the concentration of Ti(IV) in the solutions remains below 8 ppm in all cases. Although the effect of solvent in disrupting the packing of the cages in the solid state has obvious effects in the structure and accessible porosity of these family of materials (**Figure S21 and S22**), our data confirms the value of hydroxamate linkages to enable the formation of robust cages that withstand chemical degradation in a broad range of pH values.

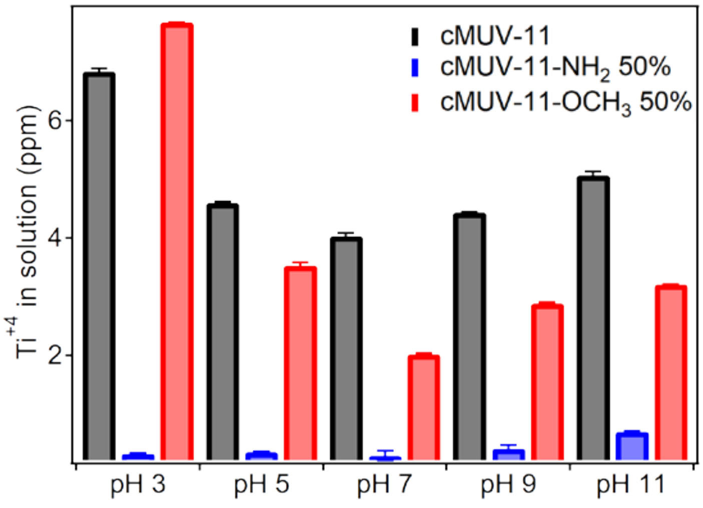

Figure S20. Titanium leaching of cMUV-11 derivatives determined by ICP-MS of the supernatant after incubation in water at variable pH for 24h (black for cMUV-11, blue for -NH_2 and red for -OCH_3).

Powder X-Ray Diffraction (PXRD) after water incubation

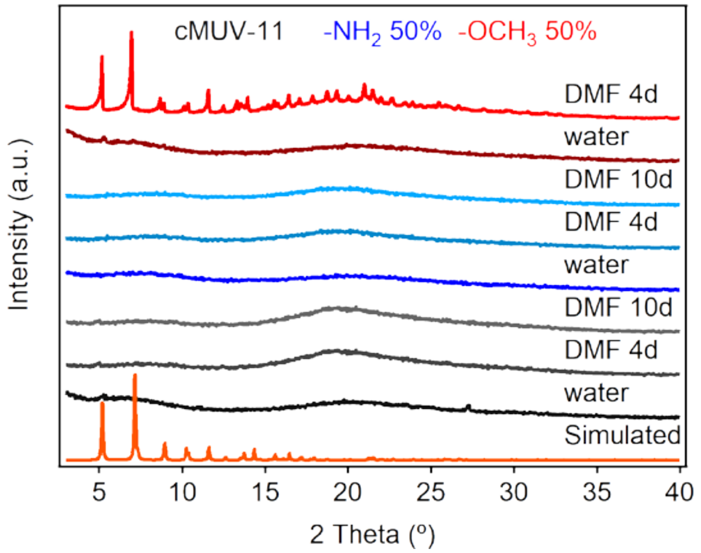

Figure S21. PXRD patterns of cMUV-11 variants (black for -H, blue for -NH2 and red for -OCH3) after water incubation for 24 hours and reversibility in DMF for 4 and 10 days.

To investigate the structural stability after water exposure, diffraction measurements of incubated solids were carried out. The PXRD pattern (**Figure S21**) of all cage variations (-H, -NH_2 and -OCH_3) confirm a structural collapse in these conditions. In this regard, we have also investigated the reversibility of this structural collapse in presence of water by soaking the solids in DMF for 4 and 10 days. As shown in **Figure S21**, only cMUV-11-OCH_3 50% recovered the original structure. We argue reversibility in this case is enabled by the hydrophobicity provided by the OCH_3 groups, that favours the regeneration of the original

structure compared to the irreversibility displayed by -H and -NH_2 variants. This is likely correlated to the smaller impact of solvent evacuation in cMUV-11-OCH_3 that permits accessing higher permanent porosities in comparable conditions of activation.

CO_2 adsorption after water incubation

We have also collected CO_2 isotherms at 298 K to investigate the effect of this structural change on the gas uptake. The cages display the relative trends in CO_2 uptake of the as-made crystals but display a significant reduction in their uptake capacity as result of the structural collapse. These changes are likely provoked by the rupture of the three-dimensional network of H-bonds that direct the packing of the cages in the solid state. The presence of water breaks the original connections and induces a random packing that results in amorphous solids with reduced uptake capacities. Overall, our results confirm the chemical stability of these hydroxamate cages, with strong joints that prevent chemical degradation in water, but the sensitivity of their packing to the presence of water. This behaviour agrees will with our original findings that anticipated an important sensitivity of the structure/porosity of the solids to solvent evacuation in presence of polar solvents

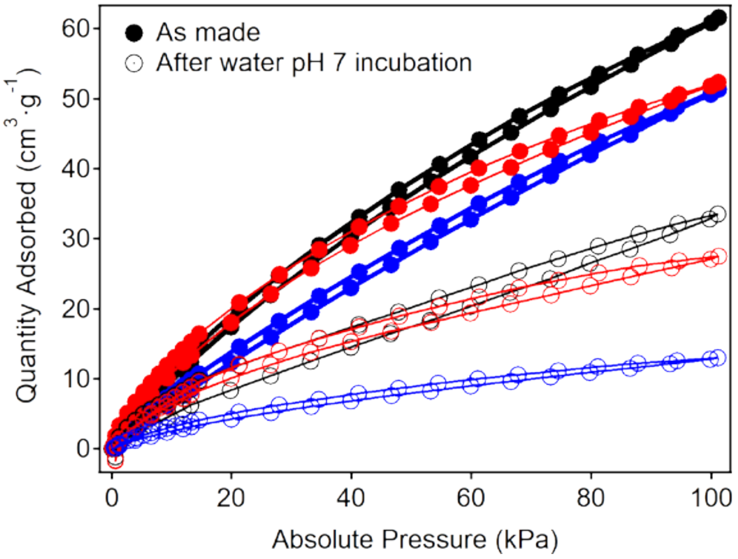

Figure S22. CO_2 adsorption/desorption isotherms of cMUV-11 (black), cMUV-11-NH_2 (blue) and cMUV-11-OCH_3 (red) cages after incubation in water (empty symbols) compared to as made crystals (filled symbols).

Scanning Electron Microscopy (SEM) after water incubation

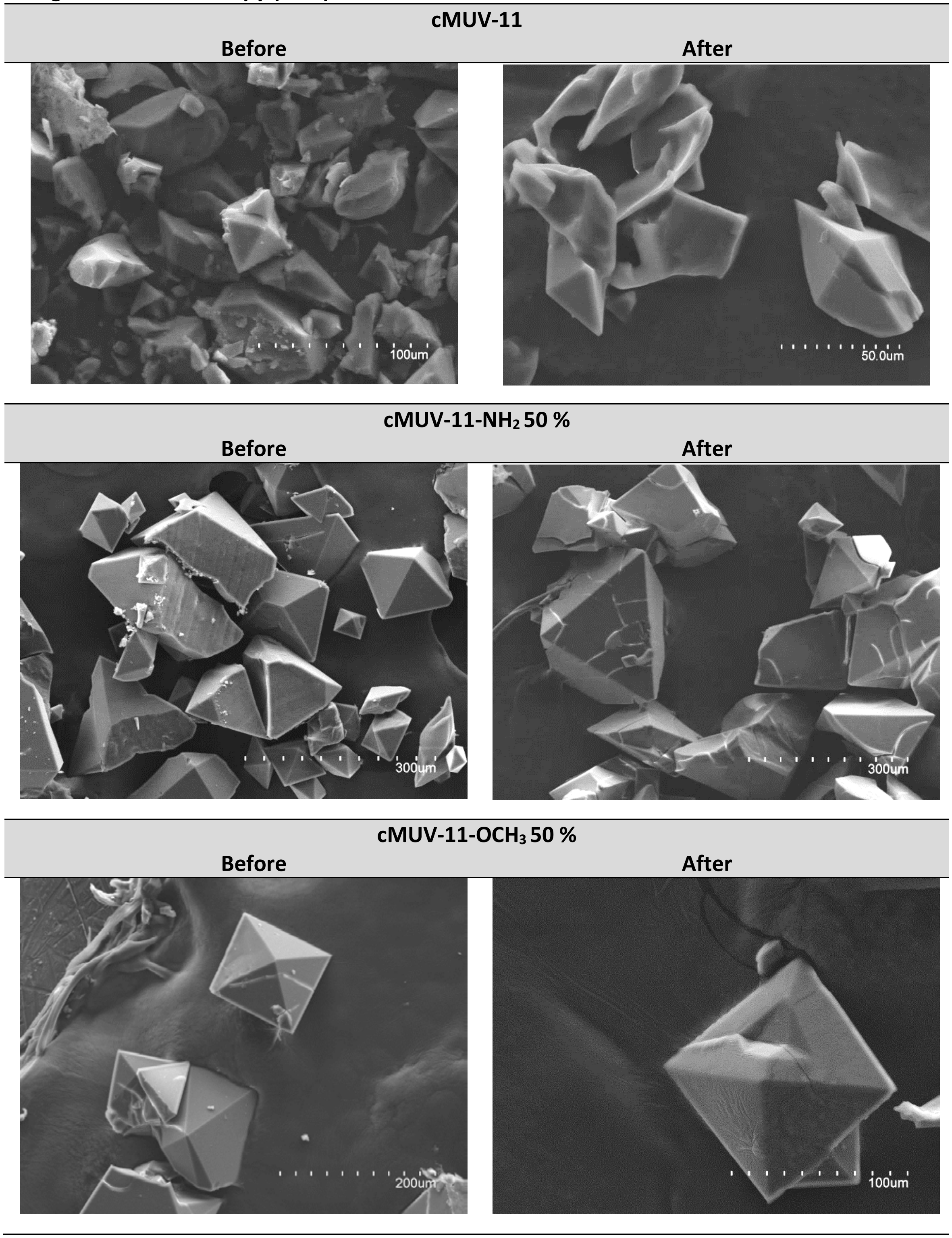

Figure S23. Scanning Electron Microscopy (SEM) images of crystals after water treatment discard significant damage during the process. For a direct comparison, images were taken from the same crystals before and after the treatment.

S7. Computational calculations

All electronic and structural calculations for each structure were calculated using DFT with VASP code[14,15] employing the screened hybrid functional HSE06[16,17] and complemented by the Tkatchenko-Scheffer dispersion method[18,19,20]. The recommended GW PAW pseudopotentials[21] were used. The kinetic energy cut-off for the plane-wave basis set expansion was chosen as 500 eV, and Γ-points were used for integrations in the reciprocal space, due to the large size of the unit cell of the direct lattice.

Hirshfeld charges for the hydroxamate group with the different substituents generated in a calculation shown in the **Figure S24**. In the interactive Hirshfeld-I method (HI),[22] the population of each atom is calculated by assuming that the charge density at each point is shared among the surrounding atoms in direct proportion to their free atom densities at the corresponding distances from the nuclei.

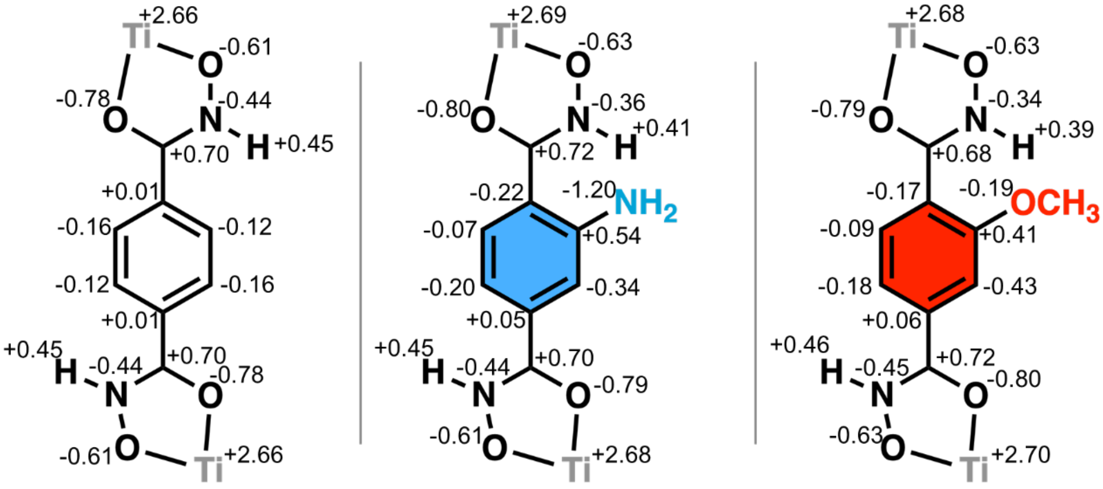

Figure S24. Hirshfeld charges for the hydroxamate group with different substituents ($|\overline{e}|$).

References

[1] Griffith D.; Krot K.; Comiskey J.; Nolan K. B.; Marmion C. J. *Dalton Trans.*, **2008**, 137–147.

[2] M. Padial, N.; Castells-Gil, J.; Almora-Barrios, N.; Romero-Angel, M.; Silva, I. da; Barawi, M.; García-Sánchez, A.; O'Shea, V. A. de la P.; Martí-Gastaldo, C. *J. Am. Chem. Soc.* **2019**, *141* (33), 13124–13133.

[3] Park H.; Kim S.; Jung B.; Park M.H.; Kim Y.; Kim M. *Inorg. Chem.* **2018**, *57*, 1040–1047

[4] Bruker. APEX3, SAINT-Plus and SADABS. Bruker AXS Inc., Madison, Wisconsin, USA. **2016**

[5] Rigaku Oxford Diffraction. CrysAlisPro Software system, version 1.171.41.112, Rigaku Corporation, Oxford, UK. **2018**

[6] Sheldrick, G.M. *Acta Cryst.* **2015**, *A71*, 3-8.

[7] Dolomanov O.V.; Bourhis L.J.; Gildea R.J; Howard J.A.K.; Puschmann H. J. *Appl. Cryst.* **2009**, *42*, 339-341.

[8] Steiner T. C–H–O Hydrogen Bonding in Crystals. *Cryst. Rev*, **1996**, *6*, 1-57

[9] Liu, G.; Yuan, Y. D.; Wang, J.; Cheng, Y.; Peh, S. B.; Wang, Y.; Qian, Y.; Dong, J.; Yuan, D.; Zhao, D. *J. Am. Chem. Soc.* **2018**, *140*, 6231-6234.

[10] Carné-Sánchez, A.; Albalad, J.; Grancha, T.; Imaz, I.; Juanhuix, J.; Larpent, P.; Furukawa, S.; Maspoch, D. *J. Am. Chem. Soc.* **2019**, *141*, 4094–4102.

[11] He, Y.-P.; Yuan, L.-B.; Chen, G.-H.; Lin, Q.-P.; Wang, F.; Zhang, L.; Zhang, J. *J. Am. Chem. Soc.* **2017**, *139*, 16845–16851.

[12] Taggart, G. A.; Antonio, A. M.; Lorzing, G. R.; Yap, G. P. A.; Bloch, E. D. *ACS Appl. Mater. Inter.* **2020**, *12*, 24913–24919.

[13] Willems, T. F.; Rycroft, C. H.; Kazi, M.; Meza, J. C.; Haranczyk, M. *Microp. Mesop. Mater.* **2012**, *149*, 134-141.

[14] Kresse G.; Furthmüller J. Efficiency of Ab-Initio Total Energy Calculations for Metals and Semiconductors Using a Plane-Wave Basis Set. *Comput. Mater. Sci*. **1996**, *6*, 15-50.

[15] Kresse G.; Furthmüller J. Efficient Iterative Schemes for Ab Initio Total-Energy Calculations Using a Plane-Wave Basis Set. Phys. Rev. B: Condens. *Matter Mater. Phys.* **1996**, *54*, 11169-11186.

[16] Heyd J.; Scuseria G. E.; Ernzerhof M. *J. Chem. Phys.*, **2003**, *118***,** 8207-8215.

[17] Krukau A. V.; Vydrov O. A.; Izmaylov A. F.; Scuseria G. E. *J. Chem. Phys.*, **2006**, *125*, 224106-1.

[19] Tkatchenko A.; Scheffler M., *Phys. Rev. Lett.,* **2009**, *102*, 073005.

[19] Bučko T.; Lebègue S.; Hafner J.; Ángyán J. G. *Phys. Rev.,* **2013**, *B 87*, 064110.

[20] Kerber; Sauer J. *J. Comp. Chem.*, **2008**, *29*, 2088.

[21] Lejaeghere K.; Bihlmayer G.; Björkman T.; Blaha P.; Blügel S. Reproducibility in density functional theory calculations of solids. *Science,* **2016**, *351*, 1415, aad3000-1.

[22] Bultinck P.; Van Alsenoy C.; Ayers P.W.; Carbó-Dorca R. *J. Chem. Phys.* **2007**, *126*, 144111.
